# Triglyceride glucose index and modified triglyceride glucose indices are instrumental to optimize 3P medical management for postpartum cardiovascular disease

**DOI:** 10.1007/s13167-026-00437-8

**Published:** 2026-02-19

**Authors:** Dong Lin, Haoxian Tang, Enoch Odame Anto, Yueran Zhou, Shenglan Zhang, Xiulian Deng, Cuihong Tian, Weijie Cao, Zhuoqiao He, Zhisheng Chen, Pengxiang Ying, Yequn Chen, Xuerui Tan

**Affiliations:** 1 Department of Cardiology, The First Affiliated Hospital of Shantou University Medical College, Shantou, 515041 Guangdong China; 2 Nutrition and Health Innovation Research Institute & School of Medical and Health Sciences, Edith Cowan University, Joondalup, Perth, 6027 Australia; 3 Department of Community Monitoring, The First Affiliated Hospital of Shantou University Medical College, Shantou, 515041 Guangdong China; 4 Institute for Glycome Study, Shantou University Medical College, Shantou, 515041 Guangdong China; 5 Joint Shantou International Eye Center of Shantou University and the Chinese University of Hong Kong, Shantou, 515041 Guangdong China; 6 Human Phenome Institute of Shantou University Medical College, Guangdong Engineering Research Centre of Human Phenome, Chemistry and Chemical Engineering Guangdong Laboratory, Shantou, 515063 Guangdong China; 7 Clinical Medical Research Center, The First Affiliated Hospital of Shantou University Medical College, Shantou, 515041 Guangdong China

**Keywords:** Hypertensive disorders of pregnancy, Postpartum cardiovascular disease, Triglyceride glucose index, Mendelian randomization, Insulin resistance, Obesity indicators, Predictive preventive personalized medicine (PPPM / 3PM), CVD prevention and management, Monitoring metabolic trajectories, Paradigm change from reactive to proactive medicine

## Abstract

**Background:**

Postpartum cardiovascular disease (CVD) following hypertensive disorders of pregnancy (HDP) involves multifactorial mechanisms linking metabolic susceptibility to vascular damage. Identifying predictive biomarkers and modifiable risk factors is crucial for early stratification in the context of predictive, preventive, and personalized medicine (PPPM). We aimed to investigate the association of triglyceride-glucose (TyG) indices with incident CVD in women with prior HDP and to assess the causal relationship using Bayesian weighted Mendelian randomization (BWMR) analysis, with a focus on the implications for PPPM strategies in postpartum management.

**Methods:**

In this longitudinal cohort study, 1,642 women with prior HDP from the UK Biobank were followed for a median of 14.05 years postpartum. TyG and modified TyG indices were assessed at enrollment. Associations with incident CVD were evaluated using Cox proportional hazards models and restricted cubic spline regression, while predictive performance was assessed with Harrell’s C-index. BWMR was further used to validate causal relationships.

**Results:**

During follow-up, 466 (28.4%) women developed postpartum CVD (mean age 51.05 ± 8.28 years). Elevated baseline TyG indices were associated with an increased risk of CVD. The highest TyG tertile had an adjusted hazard ratio (HR) of 1.35 for CVD (95% CI, 1.04–1.74; *P* = 0.023), with similar associations for modified TyG indices (adjusted HRs for the highest tertile ranging from 1.31 to 1.39 [*P* < 0.05]). C-indices were around 0.6. The stratified analysis showed more pronounced associations in younger, normotensive, and non-obese subgroups. The BWMR analysis supported a significant causal effect of genetically predicted TyG levels on CVD risk (*P* < 0.05), confirming the observational findings.

**Conclusions:**

Elevated TyG indices are significantly associated with increased postpartum CVD risk in women with prior HDP, particularly in subgroups without overt clinical risk factors. Integration of observational and genetic evidence supports a causal role of insulin resistance in this multifactorial pathology. TyG indices may serve as promising predictive biomarkers for early risk stratification and potential targets for personalized metabolic interventions, in line with PPPM strategies. Our findings highlight the importance of monitoring metabolic trajectories for effective PPPM-guided postpartum CVD prevention and management.

**Supplementary Information:**

The online version contains supplementary material available at 10.1007/s13167-026-00437-8.

## Introduction

Hypertensive disorders of pregnancy (HDP), affecting 5–10% of pregnancies worldwide, represent a significant risk factor for both maternal and fetal morbidity and mortality [1]. Emerging evidence suggests that women with HDP face a 2 to 4 fold increased risk of developing postpartum cardiovascular disease (CVD) later in life, including hypertension, coronary artery disease, and stroke [2, 3]. This elevated risk persists for decades after delivery, creating a critical window for preventive intervention that aligns with the principles of predictive, preventive, and personalized medicine (PPPM) [4].

The PPPM paradigm represents a transformative approach in healthcare, emphasizing early prediction of disease risk, targeted prevention before clinical manifestation, and personalized therapeutic strategies tailored to individual patient profiles [4, 5]. However, effective implementation requires accessible biomarkers that reflect underlying pathophysiological mechanisms and can be integrated into routine postpartum care [6]. To effectively implement the PPPM vision for postpartum women with a history of HDP, it is essential to possess a comprehensive understanding of the underlying pathophysiological connections.

The pathophysiological mechanisms linking HDP to long-term postpartum cardiovascular complications remain incompletely understood. Two predominant hypotheses have been proposed: (1) shared risk factors such as obesity and dyslipidemia may predispose to both HDP and postpartum CVD, and (2) pregnancy-specific vascular and metabolic alterations may directly contribute to persistent cardiovascular dysfunction [7, 8]. Notably, insulin resistance (IR) and metabolic dysfunction appear to play a central role in this association, potentially serving as both mediators and amplifiers of postpartum cardiovascular risk [9]. Understanding the metabolic underpinnings of this elevated risk is essential for developing predictive diagnostics and targeted interventions within the PPPM framework [10].

The triglyceride-glucose (TyG) index, calculated as *ln* [fasting triglycerides (mg/dL) × fasting glucose (mg/dL)/2], has emerged as a simple yet reliable surrogate marker of IR [11]. Recent studies have demonstrated significant associations between elevated TyG index and various cardiometabolic outcomes in general populations [12–20]. Modified TyG indices, incorporating obesity indicators such as body mass index (TyG-BMI), waist circumference (TyG-WC) and waist-to-height ratio (TyG-WHtR), have shown enhanced predictive value for cardiometabolic risk in general population [12, 19]. These composite indices may better capture the interplay between adiposity and metabolic dysfunction in cardiovascular pathogenesis [13].

Recent studies have also demonstrated the predictive value of TyG indices for CVD risk in various populations, including diabetic adults [14], general populations [15], and patients with metabolic dysfunction-associated steatotic liver disease [16]. However, the utility of TyG indices in postpartum women with prior HDP remains largely unexplored. This represents a critical knowledge gap, given the unique metabolic and vascular changes characteristic of HDP that may influence the predictive performance of these indices [17]. Furthermore, the potential differential associations of various TyG indices with specific postpartum cardiovascular outcomes in this high-risk population warrant systematic investigation.

Based on this premise, HDP can be linked to an increased long-term risk of CVD, and elevated TyG indices in this population are not merely incidental but indicate persistent insulin resistance and dyslipidemia. These metabolic disturbances contribute to endothelial dysfunction, chronic low-grade inflammation, and oxidative stress, which are fundamental drivers of atherosclerotic progression [21]. In particular, among women with a history of HDP, this metabolic environment may exacerbate arterial stiffness, promote atherogenic lipid profiles, and impair vascular repair, thereby accelerating subclinical atherosclerosis and heightening the risk of early-onset CVD [22–24]. Consequently, TyG indices may function as integrative biomarkers of residual vasculometabolic injury, linking pregnancy-related pathology with future cardiovascular outcomes [25].

TyG indices emerge as compelling candidates for integration into a PPPM approach. PPPM offers a promising framework for postpartum CVD risk management by emphasizing early prediction, tailored prevention, and individualized treatment strategies based on molecular and clinical profiling [4, 5]. In women with a history of HDP, PPPM could significantly improve long-term outcomes through timely risk stratification and personalized monitoring. However, a critical barrier remains the lack of reliable biomarkers that reflect underlying metabolic dysfunction and can be integrated into routine postpartum care [23]. TyG indices could serve as practical biomarkers for early detection, prevention, and personalized interventions, aligning with PPPM principles. In the context of PPPM, their alterations could serve as therapeutic targets for early intervention and personalized therapeutic strategies, reinforcing their relevance to PPPM.

The UK Biobank cohort, with its large sample size, comprehensive phenotypic data, long-term follow-up, and genetic data availability, provides an exceptional opportunity to address these questions within a PPPM framework. Mendelian Randomization (MR) employs genetic variants as instrumental variables (IVs) to explore causal relationships between modifiable exposures and outcomes, leveraging the random allocation of alleles during gamete formation to minimize confounding factors and reverse causation [26, 27].

### Working hypothesis in the framework of PPPM

We hypothesize that elevated TyG indices are associated with an increased risk of postpartum CVD in women with prior HDP, and that modified TyG indices provide superior predictive performance. We integrated MR analysis with the UK Biobank database for a longitudinal cohort study, thoroughly investigating the associations between the TyG index and its derived indicators (such as TyG-BMI, TyG-WC, and TyG-WHtR) and the risk of postpartum CVD in women with prior HDP. This study aims to: (1) evaluate the associations of TyG and modified TyG indices with incident postpartum CVD in women with prior HDP; (2) compare the predictive performance of different TyG indices; and (3) examine potential effect modification by key demographic and clinical characteristics. The findings may inform the development of targeted preventive interventions in line with PPPM principles for this vulnerable population, ultimately reducing their long-term postpartum cardiovascular burden.

## Methods

### Study design

**Fig. 1:**
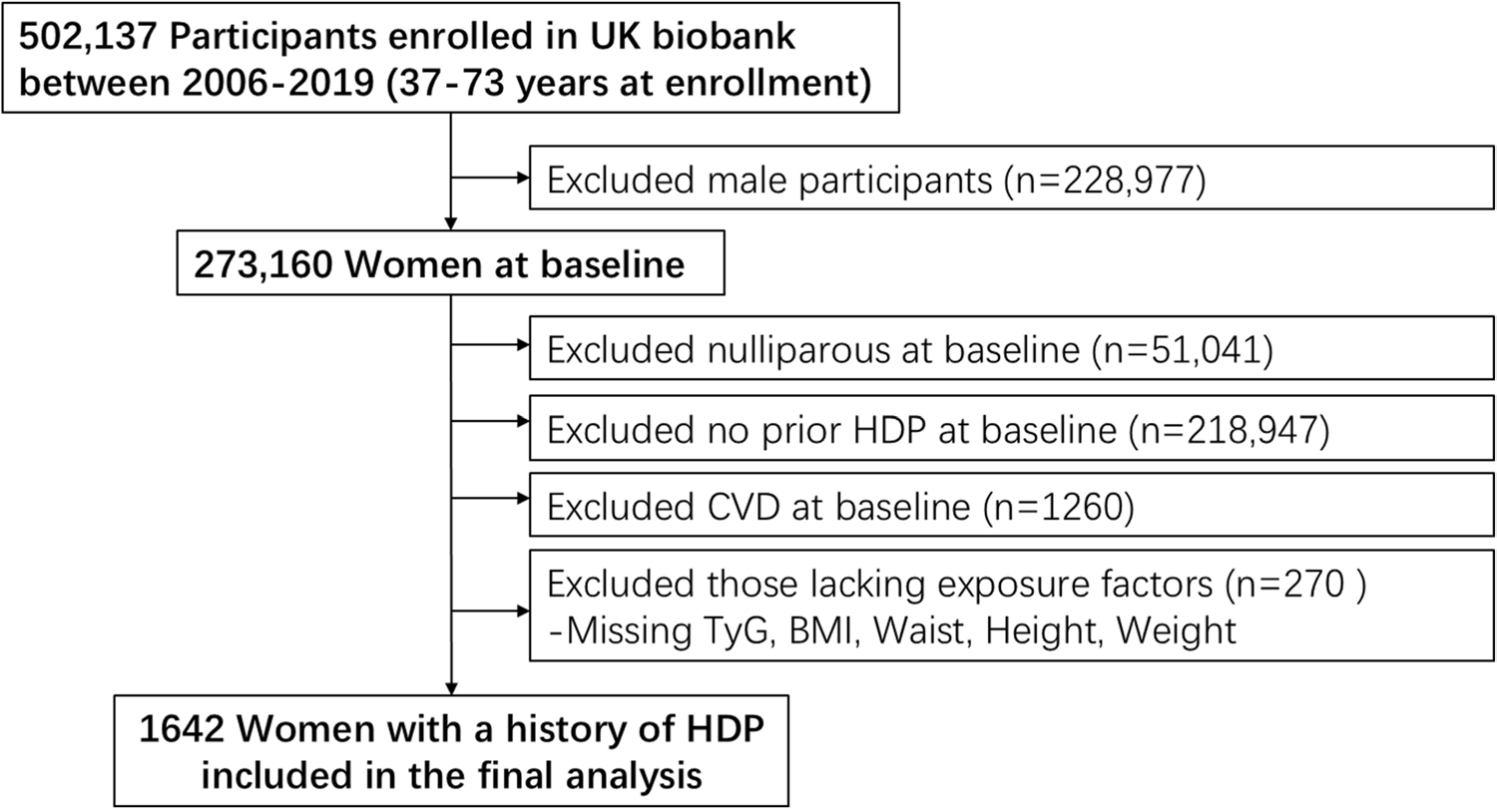
Flow chart of the population included in this study. Abbreviations: *BMI* body mass index, *CVD* cardiovascular disease, *TyG* triglyceride-glucose index, *HDP* hypertensive disorders of pregnancy

This study employed an integrated analytical framework comprising two components: (1) a longitudinal observational cohort analysis to investigate the association between baseline TyG index and modified TyG indices (TyG-BMI, TyG-WC, TyG-WHtR) with incident postpartum CVD in women with a history of HDP, and (2) a two-sample MR analysis to assess the potential causal relationship between genetically instrumented metabolic traits and CVD risk. The overall design and participant selection process are summarized in Fig. 1.

### Data sources and study population

This longitudinal cohort investigation leverages data from the UK Biobank, a population-based resource of over 500,000 individuals aged 37 to 73 years, enrolled between 2006 and 2010 across the United Kingdom. All participants provided written informed consent, completed detailed baseline assessments encompassing sociodemographic characteristics, medical history, and lifestyle factors, underwent standardized physical examinations, and donated biological specimens for biomarker analyses. The study protocol received approval from the North West Multicenter Research Ethics Committee (reference: 11/NW/03820). Analyses were performed under UK Biobank application number [205837], and reporting conforms to the Strengthening the Reporting of Observational Studies in Epidemiology (STROBE) recommendations for cohort studies.

The subjects of this current study were recruited the female participants within the UK Biobank (*n* = 273,160), inclusion criteria were as follows: (1) at least one live birth, as verified by self-reported reproductive history and hospital episode records; (2) diagnosed with HDP, including gestational hypertension or preeclampsia, ascertained via ICD-10 codes O13-O16 in hospital records or self-reported physician diagnosis; (3) availability of complete data regarding the TyG index (fasting triglycerides and fasting glucose), anthropometric indices (body mass index [BMI], waist circumference [WC], waist-to-height ratio [WHtR]), and relevant covariates. Participants were excluded if they had: (1) documented history of CVD at baseline [International Classification of Diseases, Tenth Revision (ICD-10) codes I20-I25, I60-I69]; (2) missing information on key exposures or outcome measures. The resulting analytic sample comprised 1642 women with HDP, as illustrated in Fig. 1.

### Assessment of exposure: TyG and modified TyG indices

The TyG index was computed using the formula: *ln* [fasting TG (mg/dL) × fasting glucose (mg/dL)/2]. Modified indices included: (1) TyG-BMI: TyG × BMI (kg/m^2^); (2) TyG-WC: TyG × WC (cm); (3) TyG-WHtR: TyG × (WC [cm]/height [cm]).

### Outcome definition: postpartum cardiovascular disease

The primary outcome was incident postpartum CVD, defined in accordance with the ICD-10 codes I00–I79. Participants were followed from baseline until the first documented episode of postpartum CVD, censoring at the date of death, loss to follow-up, or the end of the study period, whichever occurred first. Clinical events were identified using hospital admission diagnoses or underlying causes of death recorded in the national registry or validated self-reported physician diagnoses.

### Covariates

Potential confounders were selected a priori, guided by previous literature, and encompassed: (1) demographics: age at enrollment, age at first birth, ethnicity (categorized as White or non-White), and educational attainment (college or above vs. lower). (2) lifestyle factors: current smoking (yes vs. no), alcohol consumption (≥ 1 drink/week vs. less), and physical activity level [low, moderate, high; measured via the international Physical Activity Questionnaire (IPAQ)]. (3) clinical/metabolic factors: BMI (kg/m^2^), presence of obesity (BMI ≥ 30 kg/m^2^), systolic and diastolic blood pressure (mmHg), hypertension status (SBP ≥ 140 mmHg, DBP ≥ 90 mmHg, or antihypertensive medication use), serum lipid and profile (LDL-C, HDL-C, total cholesterol), fasting glucose, glycated hemoglobin (HbA1c), and renal function parameters [estimated Glomerular Filtration Rate (eGFR), serum creatinine]. (4) comorbidities at baseline: chronic kidney disease (CKD) (ICD-10 codes N18 or self-reported kidney dysfunction), diabetes mellitus (DM) (ICD-10 codes E10-E14 or self-reported).

### MR analysis

We employed a two-sample Mendelian Randomization (MR) design to systematically evaluate the causal effects of the TyG index and anthropometric traits on CVD risk. Summary-level genetic data were obtained from publicly available genome-wide association studies (GWAS). For the exposures, we prioritized datasets with large sample sizes to ensure statistical power. Specifically, data for the TyG index were retrieved from previous literature (http://links.lww.com/JS9/C792) [28]. Genetic instruments for BMI (ID: ieu-b-4816), WC (ID: ieu-a-61), and Waist-to-Hip Ratio (WHR, ID: ebi-a-GCST90025996) were acquired from the Integrative Epidemiology Unit (IEU) OpenGWAS database. The summary statistics for HDP were sourced from the FinnGen consortium (Freeze 11, ID: finn-b-O15_OEDEM_PROTUR_HYPERT).

To ensure the robustness and generalizability of our outcome assessment, we leveraged CVD summary statistics from two independent sources: the FinnGen study (Freeze 11, ID: finn-b-FG_CVD) and the EBI database (ID: ebi-a-GCST90029019). Because this MR analysis utilized pre-existing summary data, ethical approval and informed consent were waived.

To identify genetic variants suitable for estimating causal effects, we applied a genome-wide significance threshold of *P* < 5*10–8. To ensure independence, single nucleotide polymorphisms (SNPs) in linkage disequilibrium (R^2^ < 0.001, within 10,000 kb) were excluded. The Inverse Variance Weighted (IVW) method under a multiplicative random-effects model was employed as the primary approach to estimate causal associations, as it accounts for potential heterogeneity among instruments. Complementary methods, including MR-Egger and the Weighted Mode, were used to validate the findings.

Sensitivity analyses were conducted to ensure the robustness of the findings. Cochran’s Q statistic, and MR-Egger intercept test were applied to assess heterogeneity and potential horizontal pleiotropy among the instrumental variables. A leave-one-out sensitivity analysis was performed to verify that the results were not driven by any single influential variant. To control for multiple testing, we applied the False Discovery Rate (FDR) correction using the Benjamini–Hochberg procedure. An FDR-adjusted P-value < 0.05 was considered statistically significant.

 To further enhance the reliability of our estimates, we introduced Bayesian Weighted MR (BWMR) analysis [29]. BWMR assigns weights to each genetic instrument based on its pleiotropic effects, effectively reducing bias and providing more nuanced uncertainty quantification. 

### Statistical analyses

Descriptive statistics were used to compare baseline characteristics between women who developed postpartum CVD and those who did not during the follow-up. Continuous variables were assessed for normality using the Shapiro–Wilk test; normally distributed variables were reported as mean ± standard deviation (SD) and compared using Student’s t-test, while non-normally distributed variables were expressed as median (interquartile range, IQR) and compared using the Mann–Whitney U test. Categorical variables were summarized as frequencies (percentages) and tested using the chi-square or Fisher’s exact test as appropriate. The TyG index and its modified forms (TyG-BMI, TyG-WC and TyG-WHtR) were categorized into tertiles for analysis. Covariates were selected based on clinical relevance identified in previous literature and univariate associations with postpartum CVD (*P* < 0.1).

Kaplan–Meier curves were constructed to visualize cumulative incidence of postpartum CVD across TyG index tertiles, and log-rank tests were used to evaluate differences between groups. The associations between TyG indices and incident postpartum CVD were assessed using Cox proportional hazards regression: model 1 (unadjusted); model 2 (adjusted for age at enrollment, age at first live birth, ethnicity, education, smoking status, alcohol consumption, obesity, parity, hypertension, diabetes, and chronic kidney disease). Restricted cubic spline (RCS) regression with four knots placed at the 5^th^, 35^th^, 65^th^ and 95^th^ percentiles of the TyG indices distribution was used to examined potential non-linear relationships between continuous TyG indices and postpartum CVD risk. Linearity was assessed via likelihood ratio tests comparing linear and spline models.

Predictive performance of TyG indices for postpartum CVD was evaluated using time-dependent Harrell’s C-index, as well as the continuous net reclassification improvement (NRI) and integrated discrimination improvement (IDI), with comparisons conducted among different TyG-related metrics. Subgroup analyses were performed to investigate potential effect modification by age group, obesity, hypertension, diabetes, and chronic kidney disease at baseline; statistical interactions were assessed using Wald tests. Sensitivity analyses included further adjustment for LDL-C, eGFR, and HbA1c, and exclusion of participants with less than two years of follow-up.

All analyses were performed using R software (version 4.3.4). A two-sided *P*-value < 0.05 was considered indicative of statistical significance.

## Results

In this cohort of 1,642 women with a history of HDP, significant baseline differences were observed between women who developed incident postpartum CVD during follow-up (*n* = 466) and those who did not (*n* = 1,176) (Table 1). Women who developed postpartum CVD were significantly older at enrollment than those without postpartum CVD (mean 54.20 vs. 49.80 years; *P* < 0.001) and had a younger age at first live birth (27.31 vs. 29.39 years; *P* < 0.001). The prevalence of multiple cardiovascular risk factors was also higher in the postpartum CVD group, including obesity (31.55% vs. 22.36%, *P* < 0.001), baseline diabetes (6.88% vs. 3.76%,* P* = 0.007), and hypertension (82.34% vs. 61.43%, *P* < 0.001). In contrast, the prevalence of higher education (46.24% vs. 51.92%, *P* = 0.038) and alcohol consumption (58.49% vs. 64.26%, *P* = 0.03) was lower among women who developed postpartum CVD. Anthropometric and metabolic parameters differed substantially, women with postpartum CVD had higher BMI (28.42 vs. 26.91 kg/m^2^), lower eGFR (96.10 vs. 102.34 ml/min/1.73m^2^), higher LDL-C (3.67 vs. 3.51 mmol/L), and increased HbA1c (35.59% vs. 34.37%; all *P* < 0.001) compared to those without postpartum CVD. Notably, all TyG indices (TyG, TyG-BMI, TyG-WHtR and TyG-WC) were significantly elevated in postpartum CVD cases compared to controls (*P* < 0.001), with higher tertiles of each index consistently associated with increased postpartum CVD risk.

**Table 1:** Baseline characteristics according to postpartum CVD outcome

Characteristics	Total	Postpartum CVD incidence		*P*-value
		No	Yes	
Number of participants	1642	1176	466	
Age	51.05 ± 8.28	49.80 ± 7.69	54.20 ± 8.87	< 0.001^***^
Age at first live birth	28.80 ± 6.12	29.39 ± 6.06	27.31 ± 6.01	< 0.001^***^
Age at menopause last menstrual period	49.42 ± 5.32	49.35 ± 5.36	49.54 ± 5.25	0.681
Race, White (%)				0.598
Yes	1561 (95.47)	1116 (95.30)	445 (95.91)	
No	74 (4.53)	55 (4.70)	19 (4.09)	
Higher education (%)				0.038^*^
Yes	822 (50.31)	607 (51.92)	215 (46.24)	
No	812 (49.69)	562 (48.08)	250 (53.76)	
Obesity (%)				< 0.001^***^
Yes	410 (24.97)	263 (22.36)	147 (31.55)	
No	1232 (75.03)	913 (77.64)	319 (68.45)	
Current smoking (%)				0.083
Yes	119 (7.26)	77 (6.56)	42 (9.03)	
No	1519 (92.74)	1096 (93.44)	423 (90.97)	
Current drinking (%)				0.03^*^
Yes	1027 (62.62)	755 (64.26)	272 (58.49)	
No	613 (37.38)	420 (35.74)	193 (41.51)	
Multiple live birth (%)				0.565
Yes	27 (1.64)	18 (1.53)	9 (1.93)	
No	1615 (98.36)	1158 (98.47)	457 (98.07)	
Diabetes at baseline (%)				0.007^**^
Yes	76 (4.65)	44 (3.76)	32 (6.88)	
No	1560 (95.35)	1127 (96.24)	433 (93.12)	
Chronic kidney disease at baseline (%)				0.239
Yes	6 (0.37)	3 (0.26)	3 (0.64)	
No	1636 (99.63)	1173 (99.74)	463 (99.36)	
Hypertension at baseline (%)				< 0.001^***^
Yes	1077 (67.35)	704 (61.43)	373 (82.34)	
No	522 (32.65)	442 (38.57)	80 (17.66)	
BMI, kg/m^2^	27.34 ± 5.21	26.91 ± 5.11	28.42 ± 5.32	< 0.001^***^
eGFR, ml/min/1.73m^2^	100.57 ± 13.66	102.34 ± 12.69	96.10 ± 14.97	< 0.001^***^
LDL, mmol/L	3.56 ± 0.82	3.51 ± 0.81	3.67 ± 0.84	< 0.001^***^
HbA1c, mmol/mol	34.72 ± 5.41	34.37 ± 4.68	35.59 ± 6.84	< 0.001^***^
TyG	1.14 ± 0.52	1.09 ± 0.50	1.26 ± 0.53	< 0.001^***^
TyG-BMI	32.05 ± 17.65	30.20 ± 16.77	36.71 ± 18.94	< 0.001^***^
TyG-WHtR	60.78 ± 32.67	57.33 ± 30.89	69.50 ± 35.33	< 0.001^***^
TyG-WC	99.16 ± 52.72	93.71 ± 49.95	112.91 ± 56.93	< 0.001^***^
TyG tertile				< 0.001^***^
Low	679 (41.35)	531 (45.15)	148 (31.76)	
Middle	530 (32.28)	370 (31.46)	160 (34.33)	
High	433 (26.37)	275 (23.38)	158 (33.91)	
TyG-BMI tertile				< 0.001^***^
Low	653 (39.77)	512 (43.54)	141 (30.26)	
Middle	529 (32.22)	379 (32.23)	150 (32.19)	
High	460 (28.01)	285 (24.23)	175 (37.55)	
TyG-WHtR				< 0.001^***^
Low	665 (40.50)	522 (44.39)	143 (30.69)	
Middle	541 (32.95)	380 (32.31)	161 (34.55)	
High	436 (26.55)	274 (23.30)	162 (34.76)	
TyG-WC tertile				< 0.001^***^
Low	660 (40.19)	514 (43.71)	146 (31.33)	
Middle	541 (32.95)	387 (32.91)	154 (33.05)	
High	441 (26.86)	275 (23.38)	166 (35.62)	

*BMI* body mass index, *CVD* cardiovascular disease, *eGFR* estimated glomerular filtration rate, *LDL-C* low-density lipoprotein cholesterol, *HbA1c* haemoglobin A1c, *TyG* Triglyceride-glucose index, *WC* Waist circumference, *WHtR* Waist-to-height ratio

The results are presented as the mean ± SD or n (%). The t-test was used for continuous variables, and the chi-square test was used for categorical variables. Higher education was defined as college education or above. Obesity was defined as body mass index (BMI) ≥ 30 kg/m^2^. Current smoking was defined as self-reported smoking at the time of assessment. Current drinking was defined as alcohol consumption at least once per week. Multiple live birth was defined as history of ≥ 5 live births

* *P* < 0.05, ** *P* < 0.01, *** P < 0.001

Table 2 shows the associations between TyG indices and incident postpartum CVD. After multivariable adjustment, the TyG index demonstrated a significant association with incident CVD, with a HR of 1.22 per 1-SD increase (95% CI, 1.01–1.49). For tertile-based analysis, the highest TyG tertile showed an HR of 1.35 (95% CI, 1.04–1.74) compared to the lowest tertile. Similar patterns were observed for modified TyG indices: TyG-BMI (highest tertile HR, 1.39; 95% CI, 1.06–1.82), TyG-WHtR (highest tertile HR, 1.31; 95% CI, 1.01–1.71), and TyG-WC (highest tertile HR, 1.33; 95% CI 1.02–1.72). All continuous and categorical associations remained statistically significant (*P* < 0.05) after adjustment for age at enrolment, age at first live birth, race, education, smoking, alcohol consumption, obesity, multiple births, hypertension, diabetes, and chronic kidney disease.

**Table 2:** Associations of TyG index and modified indices with postpartum CVD onset

	Crude		Adjusted Model	
	HR (95% CI)	*P*-value	HR (95% CI)	*P*-value
TyG (per 1 SD)	1.70 (1.44, 2.01)	< 0.001^***^	1.22 (1.01, 1.49)	0.041^*^
TyG Tertile				
Low	ref		ref	
Middle	1.47 (1.15, 1.88)	0.002^**^	1.25 (0.97, 1.61)	0.084
High	2.00 (1.58, 2.52)	< 0.001^***^	1.35 (1.04, 1.74)	0.023^*^
TyG-BMI (per 1 SD)	1.02 (1.01, 1.02)	< 0.001^***^	1.01 (1.00, 1.02)	0.006^**^
TyG-BMI Tertile				
Low	ref		ref	
Middle	1.44 (1.13, 1.84)	0.003^**^	1.12 (0.87, 1.45)	0.383
High	2.06 (1.64, 2.60)	< 0.001^***^	1.39 (1.06, 1.82)	0.017^*^
TyG-WHtR (per 1 SD)	1.01 (1.01, 1.01)	< 0.001^***^	1.00 (1.00, 1.01)	0.007^**^
TyG-WHtR Tertile				
Low	ref		ref	
Middle	1.42 (1.11, 1.81)	0.005^**^	1.15 (0.89, 1.49)	0.270
High	2.00 (1.58, 2.52)	< 0.001^***^	1.31 (1.01, 1.71)	0.044^*^
TyG-WC (per 1 SD)	1.01 (1.00, 1.01)	< 0.001^***^	1.00 (1.00, 1.00)	0.008^**^
TyG-WC Tertile				
Low	ref		ref	
Middle	1.28 (1.00, 1.64)	0.046^*^	1.06 (0.82, 1.37)	0.631
High	1.97 (1.57, 2.47)	< 0.001^***^	1.33 (1.02, 1.72)	0.033^*^

*BMI* body mass index, *CI* confidence interval, *HR* hazard ratio, *SD* standard deviation, *TyG* Triglyceride-glucose index, *WC* Waist circumference, *WHtR* Waist-to-height ratio. Tertile categories (Low, Middle, High) are based on sample distribution

Model adjusted for age at enrolment, age at first live birth, race, education levels, current smoking, current drinking, obesity, multiple live birth, history of hypertension, diabetes and chronic kidney disease. * *P* < 0.05, ** *P* < 0.01, *** P < 0.001

**Fig. 2:**
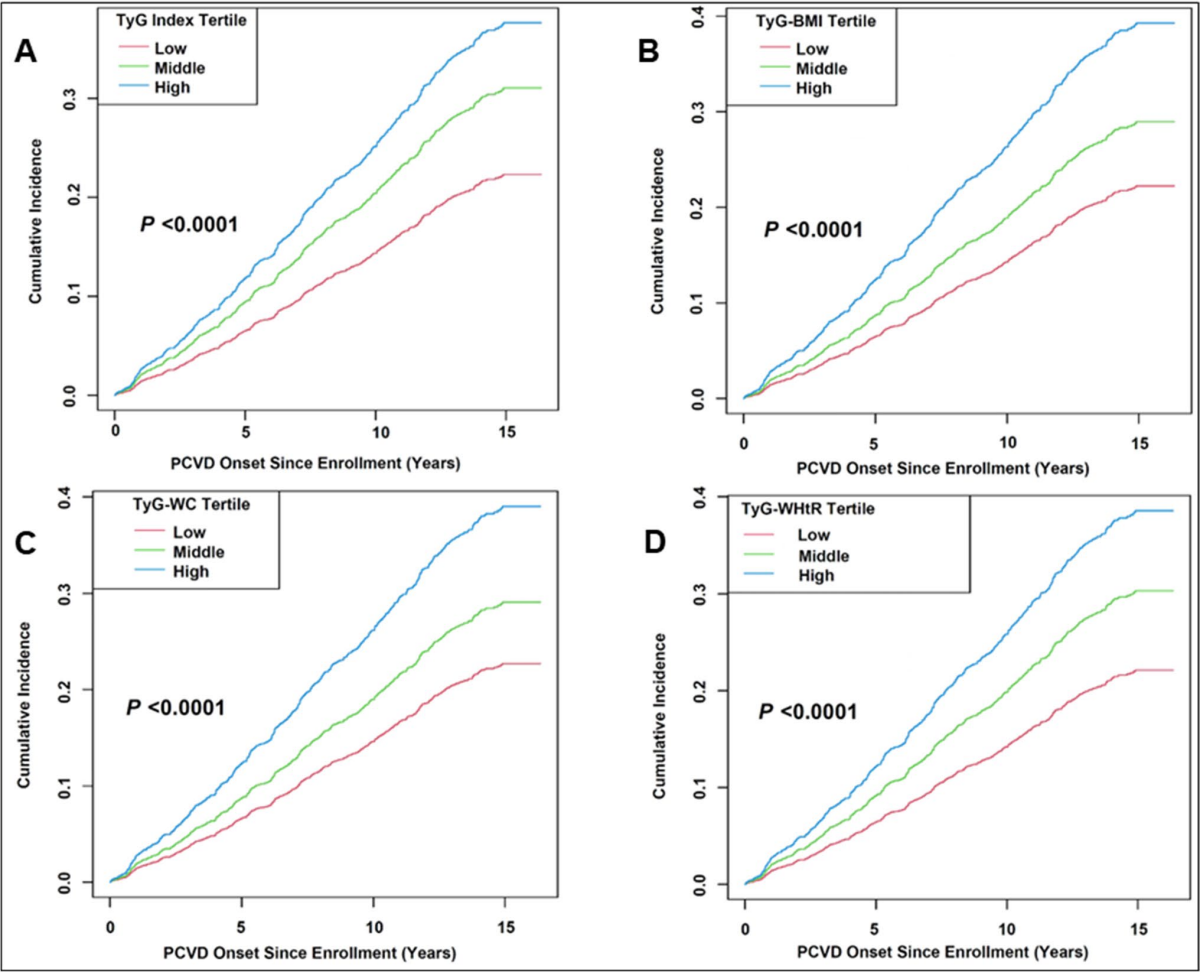
Cumulative incidence of postpartum cardiovascular disease by TyG indices tertiles. Abbreviations: *BMI* body mass index, *PCVD* postpartum cardiovascular disease, *TyG* triglyceride-glucose index, *WC* waist circumference, *WHtR* waist-to-height ratio. Note: Kaplan–Meier curves showing cumulative incidence of postpartum cardiovascular disease stratified by tertiles (Low, Middle, High) of: (**A**) TyG index; (**B**) TyG-BMI; (**C**) TyG-WC; and (**D**) TyG-WHtR among women with prior hypertensive disorders of pregnancy. *P*-values were calculated using the log-rank test

Figure 2 presents the cumulative incidence of postpartum CVD over time according to tertiles of TyG-related indices (TyG, TyG-WHtR, TyG-BMI and TyG-WC). Across all four indices, the high tertile group consistently exhibited the highest cumulative incidence of postpartum CVD throughout the follow-up period, followed by the middle and low tertile groups. The log-rank test revealed statistically significant differences among tertiles for each index (*P* < 0.0001).

RCS models demonstrated the dose–response relationships between TyG index and its modified indices with the incidence of postpartum CVD (Supplementary Fig. S1). After adjusting for covariates, all TyG indices showed a significant overall linear association with CVD risk (all *P* for overall < 0.05, and *P* for non-linear > 0.05), indicating a positive linear association without evidence of non-linearity.

Time-dependent receiver operating characteristic (ROC) analysis was employed to assess the predictive performance of TyG index and its modifications over time (Supplementary Fig. S2). The C-indices for predicting postpartum CVD onset at different time points (ranging from 6 to 14 years) were generally stable for all four indices, with overall values being approximately 0.6.

Subgroup analyses showed significant positive associations between TyG indices and postpartum CVD risk across both age groups (< 49 and ≥ 49 years), in both obese and non-obese women, and in participants with or without baseline hypertension (all *P* < 0.05, Table 3). The association was more pronounced in younger and non-hypertensive subgroups. No significant association was observed in participants with baseline diabetes, which may be attributable to the limited sample size in this subgroup. All models were adjusted for age of first live birth, race, education levels, current smoking status, current drinking status, and multiple live birth (Table 3). Sensitivity analyses showed that higher TyG and its related indices were consistently associated with increased postpartum CVD risk, both as continuous and categorical variables (Supplementary Table S1). The highest tertile groups exhibited significantly higher risks compared to the lowest. These associations remained statistically significant after adjusting for multiple confounders and after excluding participants with short follow-up, indicating the robustness of the results. TyG-WHtR and TyG-WC demonstrated significant improvements in IDI over TyG index (*p* < 0.05), while TyG-BMI did not (Supplementary Table S2). No significant differences in NRI were observed among all indices comparisons. These findings suggest modest but statistically significant discrimination improvements for modified TyG indices incorporating abdominal obesity measures.

**Table 3:** Subgroup analyses of the relationship between TyG index and modified indices with postpartum CVD incidence in a population with HDP

	Number of participants	TyG		TyG-BMI		TyG-WHtR		TyG-WC	
		HR (95% CI)	*P*-value	HR (95% CI)	*P*-value	HR (95% CI)	*P*-value	HR (95% CI)	*P*-value
Age group									
< 49	792	1.68 (1.26, 2.24)	< 0.001^***^	1.02 (1.01, 1.02)	< 0.001^***^	1.01 (1.00, 1.01)	< 0.001^***^	1.01 (1.00, 1.01)	< 0.001^***^
≥ 49	850	1.30 (1.04, 1.64)	0.021^*^	1.01 (1.00, 1.02)	0.002^**^	1.01 (1.00, 1.01)	0.001^***^	1.00 (1.00, 1.01)	0.002^**^
Obesity									
Yes	410	1.40 (1.04, 1.89)	0.026^*^	1.01 (1.00, 1.02)	0.025^*^	1.01 (1.00, 1.01)	0.009^**^	1.00 (1.00, 1.01)	0.013^*^
No	1232	1.52 (1.21, 1.91)	< 0.001^***^	1.02 (1.01, 1.03)	< 0.001^***^	1.01 (1.00, 1.01)	< 0.001^***^	1.01 (1.00, 1.01)	< 0.001^***^
Hypertension at baseline									
Yes	1077	1.39 (1.14, 1.69)	0.001^***^	1.01 (1.01, 1.02)	< 0.001^***^	1.01 (1.00, 1.01)	< 0.001^***^	1.00 (1.00, 1.01)	< 0.001^***^
No	522	1.88 (1.25, 2.82)	0.002^**^	1.02 (1.01, 1.03)	< 0.001^***^	1.01 (1.00, 1.02)	< 0.001^***^	1.01 (1.00, 1.01)	< 0.001^***^
Diabetes at baseline									
Yes	76	1.41 (0.87, 2.29)	0.168	1.01 (0.99, 1.02)	0.238	1.00 (1.00, 1.01)	0.189	1.00 (1.00, 1.01)	0.180
No	1560	1.45 (1.20, 1.76)	< 0.001^***^	1.01 (1.01, 1.02)	< 0.001^***^	1.01 (1.00, 1.01)	< 0.001^***^	1.00 (1.00, 1.01)	< 0.001^***^
CKD at baseline									
Yes	6	NA		NA		NA		NA	
No	1636	1.55 (1.30, 1.84)	< 0.001^***^	1.01 (1.01, 1.02)	< 0.001^***^	1.01 (1.01, 1.01)	< 0.001^***^	1.00 (1.00, 1.01)	< 0.001^***^

*BMI* body mass index, *CI* confidence interval, *CKD* chronic kidney disease, *CVD* cardiovascular disease, *eGFR* estimated glomerular filtration rate, *HDP* hypertensive disorders of pregnancy, *HR* hazard ratio, *TyG* Triglyceride-glucose index, *WC* Waist circumference, *WHtR* Waist-to-height ratio

Adjusted for age of first live birth, race, education levels, current smoking status, current drinking status, and multiple live birth. NA (not available): hazard ratio not calculated due to low event number in the CKD at baseline subgroup. * *P* < 0.05, ** *P* < 0.01, *** P < 0.001

### Genetic evidence

**Fig. 3:**
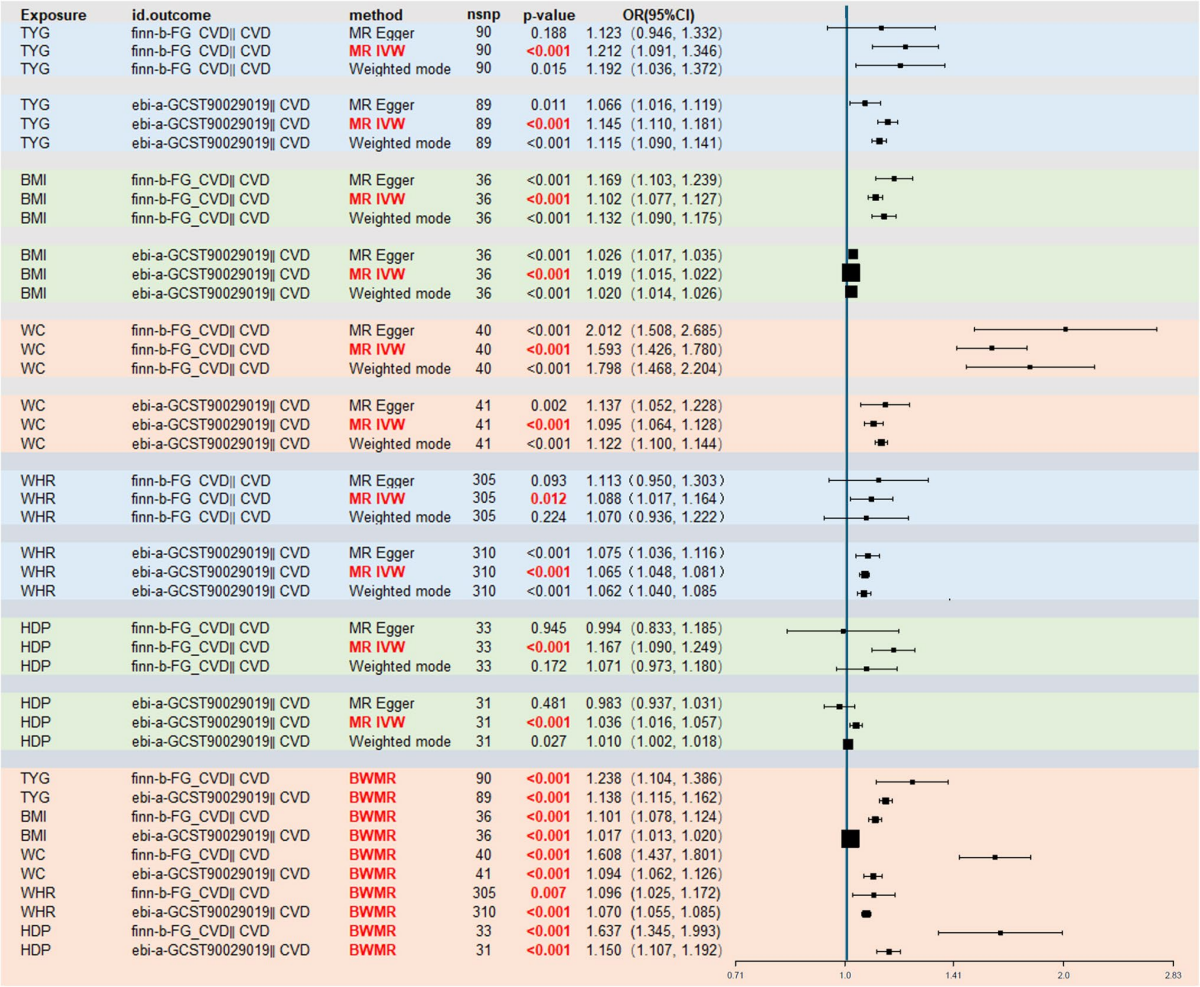
Forest plot of genetic association models. Abbreviations: BMI, body mass index; BWMR, Bayesian weighted Mendelian randomization; CI, confidence interval; CVD, cardiovascular disease; HDP, hypertensive disorders of pregnancy; IVW, inverse variance weighted; MR, Mendelian randomization; OR, odds ratio; TyG, triglyceride-glucose; WHR, waist-to-hip ratio; WC, waist circumference

Figures 3 provide robust genetic evidence for significant causal associations between the TyG index, anthropometric traits, and HDP with CVD risk across two independent GWAS datasets. Specifically, the IVW analysis identified a significant causal effect of the TyG index on CVD in both the FinnGen (OR = 1.212, 95% CI: 1.091–1.346, *P* < 0.001) and EBI datasets (OR = 1.145, 95% CI: 1.110–1.181, *P* < 0.001). Similar positive associations were observed for BMI, WC, WHR, and HDP. To address potential false positives arising from multiple testing, we applied false discovery rate (FDR) correction using the Benjamini–Hochberg procedure. All primary IVW associations remained statistically significant after adjustment, underscoring the reliability of our findings (Supplementary Table S4).

To further evaluate the robustness of theses associations, we performed several sensitivity analyses and visualizations. The scatter plots (Supplementary Fig. S3) visualizing the SNP-exposure and SNP-outcome effects generally displayed an upward slope, consistent with the positive associations of TyG, anthropometric traits, HDP with CVD risk. Cochran’s Q test indicated the presence of heterogeneity in the analysis of TyG indices and CVD (Supplementary Table S5); therefore, random-effects IVW model were applied, yielding results consistent with the primary analyses (Fig. 3). MR-Egger intercept tests suggested no evidence of horizontal pleiotropy for most associations (*P* > 0.05), although potential pleiotropy was detected for the TyG index in the EBI dataset and BMI in the FinnGen dataset (Supplementary Table S5). Leave-one-out analyses (Supplementary Fig. S4) demonstrated that no single SNP disproportionately influenced the overall results, indicating the stability of our MR estimates.

To further address potential horizontal pleiotropy and enhance the robustness of our findings, we applied BWMR, which explicitly models pleiotropy. The BWMR analysis consistently confirmed significant associations across all exposures and outcomes (all *P* < 0.001, Fig. 3), thereby reinforcing the stability of our primary results. These findings collectively suggest that a genetically predicted higher TyG index, greater adiposity, and a history of HDP are causally associated with an increased risk of CVD.

## Discussion

From the perspective of PPPM, clarifying the relationship between metabolic dysfunction (captured by the TyG index and its derivatives) and postpartum CVD is essential for early prediction of individual risk trajectories, targeted prevention focused on modifiable metabolic pathways, and personalized interventions aligned with patient-specific risk profiles [30, 31]. In this longitudinal cohort of 1,642 women with a history of HDP from the UK Biobank, our findings advance each of these objectives: (1) all TyG indices were significantly higher in women who developed postpartum CVD, with the highest tertiles associated with markedly increased risk (adjusted HRs: 1.31–1.39); (2) RCS analyses demonstrated linear dose–response relationships between TyG indices and postpartum CVD risk; and (3) these associations remained robust across strata defined by age, obesity status, and baseline hypertension. To our knowledge, this is the first study to systematically integrate observational and genetic evidence, identifying TyG indices as both predictive biomarkers and potential causal mediators of postpartum CVD risk in this high-risk group and thereby providing a foundation for PPPM-guided postpartum cardiovascular care.

The interplay among insulin resistance, visceral adiposity, and CVD risk in women with prior HDP is an area of increasing mechanistic and clinical interest. Multiple studies have explored these associations [2, 23, 32], with Tschiderer et al. [33] employing Mendelian randomization to demonstrate that genetic susceptibility to HDP correlates with unfavorable cardiovascular profiles, while Groenhof et al. [34] documented heightened risk of developing hypertension later in life among women with prior HDP. While previous work has established HDP as an important marker of future hypertension and CVD [23, 31, 34], our study extends this paradigm by demonstrating that TyG indices, which jointly reflect insulin resistance and adverse adiposity, serve as quantifiable, potentially modifiable risk factors spanning the continuum from pregnancy to long-term cardiovascular health. By documenting robust temporal associations between baseline TyG indices and incident CVD events years after delivery, our results offer new PPPM-aligned insights for risk prediction and targeted prevention. Notably, the predictive effect of the TyG index was particularly prominent in subgroups typically considered clinically low-risk, including younger women, individuals with normal baseline blood pressure, and non-obese subjects. This pattern suggests that the TyG index may capture early compensatory metabolic dysfunction, characterized by adverse visceral fat distribution and subclinical inflammation, even before overt obesity or hypertension becomes apparent. This phenomenon aligns closely with the "insulin resistance (IR) visceral obesity" axis [35], where insulin resistance impairs endothelial nitric oxide synthase (eNOS) activity, reducing nitric oxide bioavailability, while visceral adiposity promotes the secretion of pro-inflammatory cytokines, creating a pro-oxidant, pro-inflammatory state [36, 37]. This axis synergizes with HDP-induced placental ischemia and oxidative stress pathways, thereby establishing a persistent foundation for postpartum cardiovascular risk, characterized by sustained endothelial dysfunction and vascular remodeling that are fundamental to the pathogenesis of CVD after HDP [38–40].

Wu et al. [41] in their systematic review and meta-analysis, established preeclampsia as a significant predictor of future cardiovascular morbidity and underscored the need for better predictive markers in postpartum care. Addressing this need, our study investigated the TyG index as a quantifiable metabolic indicator in women with prior HDP. To complement observational findings and strengthen causal inference, we further performed MR analysis. The IVW method revealed a significant positive causal association between the TyG index and overall CVD risk. Importantly, to address potential pleiotropy, we introduced BWMR; its results further supported a causal role of TyG‑related metabolic dysregulation in CVD pathogenesis. The concordance between longitudinal cohort analysis and genetic causal inference substantially strengthens the hypothesis that the TyG index is not merely a correlative marker but an active participant in postpartum CVD development, thereby solidifying its clinical relevance as a target for PPPM strategies.

In contrast to some prior studies in general populations that have reported non-linear associations between metabolic indices and CVD [42, 43], our analyses consistently identified linear dose–response relationships between TyG indices and postpartum CVD among women with prior HDP. This linearity, observed in both continuous and tertile-based analyses, reinforces the clinical utility of TyG indices for risk stratification. The consistency of findings across observational and genetic analyses (BWMR) strengthens causal inference. Notably, their predictive strength was particularly evident in subgroups often regarded as lower risk in routine practice, namely younger women (< 49 years; HR, 1.68 vs. 1.30 in older women), those normotensive at baseline (HR, 1.88 vs. 1.39 in hypertensive women), and non-obese individuals (HR, 1.52 vs. 1.40 in obese women). This pattern suggests that TyG indices may detect early, compensatory metabolic derangements, driven by unfavorable visceral fat distribution and low-grade inflammation [44, 45], before overt obesity or hypertension emerges, in line with the PPPM focus on identifying suboptimal health states amenable to early intervention. Notably, we observed consistent associations across age and obesity subgroups (all *P* < 0.05), aligning with findings from Hong et al. [46] in patients with cardiovascular-kidney-metabolic (CKM) syndrome. In contrast, no significant association was detected in the small diabetic subgroup (*n* = 76), which most likely reflects limited statistical power rather than a true null effect. This finding contrasts with the U-shaped relationship reported in diabetic populations by Liu et al. [43] and underscores the need for further research in this specific population.

Our findings reinforce the role of TyG indices as practical, low-cost tools for postpartum cardiovascular risk stratification. As continuous surrogate markers of insulin resistance and adverse visceral adiposity, TyG indices enable quantitative identification of subclinical cardiometabolic risk beyond traditional categorical classifications based solely on HDP status. The robust predictive value maintained throughout our extended follow-up period (6–14 years) further strengthens the clinical relevance of these findings, suggesting that metabolic alterations captured by TyG indices may represent enduring pathophysiological changes rather than transient postpartum adaptations. Regular TyG indices screening may enhance postpartum risk stratification, particularly among seemingly low‑risk women (younger, normotensive, non‑obese), a group in whom conventional risk‑based follow‑up is often limited despite accumulating evidence of elevated long‑term CVD susceptibility [47–49]. Women with adverse metabolic profiles may benefit from lifestyle interventions targeting glycemic control and visceral adiposity, strategies that have been shown to improve insulin sensitivity and cardiometabolic outcomes in postpartum and high‑risk populations [50], with pharmacological approaches considered for persistent cases [51]. Integrating TyG indices into structured postpartum monitoring protocols, beginning with early metabolic assessment, may facilitate timely and individualized intervention [41]. This strategy directly promotes the PPPM goal of shifting from reactive management to proactive, biomarker-informed prevention based on each woman’s biological profile. While these strategies require validation through interventional studies, they provide a foundation for evidence based, biomarker informed protocols to reduce cardiovascular risk in this vulnerable population.

### Strengths and Limitations

This study has several strengths. It draws on a large, well-characterized longitudinal cohort to establish temporal relationships between TyG indices and postpartum CVD. A key methodological strength of this study lies in the integration of observational analysis with MR, particularly through the application of BWMR to reinforce causal inference, thereby providing a more comprehensive and multi‑layered evidence base. The consistent linear dose–response patterns observed across multiple TyG derivatives enhance the robustness and reproducibility of the findings. In addition, framing the analyses within a PPPM paradigm provides a clear translational pathway from epidemiologic evidence to clinical risk prediction, targeted prevention, and personalized postpartum care.

Limitations warrant consideration when interpreting our findings. First, reliance on self-reported HDP history without standardized obstetric confirmation may have introduced misclassification, likely biasing estimates toward the null. Second, metabolic parameters were measured at a single post-enrollment timepoint, limiting our ability to characterize detailed longitudinal trajectories of metabolic recovery or deterioration after pregnancy. Third, generalizability may be constrained by the characteristics of the UK Biobank cohort, including older recruitment age (37–73 years) and predominantly European ancestry, which may not fully reflect younger, more ethnically diverse postpartum populations. Finally, the lack of detailed data on placental pathology, body fat distribution, and longitudinal lipid profiles before, during, and after pregnancy restricts mechanistic interpretation. Despite these limitations, the large sample size and the robust, consistent associations we observed between TyG indices and long-term postpartum CVD risk underscore their potential as clinically relevant indicators of persistent metabolic dysfunction.

These limitations highlight the need for future prospective studies with longitudinal metabolic profiling from early pregnancy onwards in diverse, younger populations. Validating these findings in such cohorts and integrating them with placental pathology and detailed body composition data will be crucial to elucidate the complete trajectory and mechanisms linking HDP to future cardiometabolic risk.

### Conclusion and expert recommendations in the framework of 3P medicine

In conclusion, in the framework of PPPM, our study demonstrates a clear, linear dose–response relationship between TyG indices and incident postpartum CVD in women with a history of HDP, supported by both observational and genetic causal evidence. TyG derivatives, particularly TyG-BMI, TyG-WC, and TyG-WHtR, provide effective risk stratification, even among women conventionally considered low risk, by revealing latent metabolic dysfunction rooted in the insulin resistance–visceral adiposity axis. Leveraging TyG indices could enhance early detection, facilitate risk stratification, and support primary prevention efforts for postpartum CVD. These observations support a shift away from uniform postpartum follow-up toward a stratified, biomarker-driven prevention strategy. Further research in diverse populations with standardized obstetric phenotyping and longitudinal assessment is warranted to better understand these relationships.

### Predictive medical approaches

The core of predictive medicine lies in identifying individuals in a suboptimal health status or pre-disease state [28, 29, 52, 53]. HDP acts as a powerful metabolic stress test that unmasks underlying cardiometabolic vulnerability. Within the PPPM framework, the central predictive question is not merely who experienced HDP, but who exhibit persistent metabolic dysfunction after pregnancy. Our findings demonstrate that the TyG indices offer a quantitative means to answer this question, identifying women in a suboptimal health state long before clinical CVD appears [54]. After delivery, metabolic trajectories of women with a history of HDP diverge: some women recover to normal, while others maintain persistent dysfunction, potentially leading to irreversible pathophysiological changes and future clinical cardiovascular disease. Incorporating TyG indices into postpartum assessments enables early risk stratification and creates a critical window for timely intervention before overt postpartum CVD occurs.

### Targeted prevention

Traditional preventive strategies often apply uniform interventions to all women with a history of HDP, overlooking fundamental differences in metabolic recovery. Under the guidance of the PPPM paradigm [52], TyG indices enable graded, risk-adapted prevention for stratifying metabolic risk in postpartum women, revealing not only fundamental biological variability but also distinguishing between metabolic recovery and persistent dysfunction. The causal evidence provided by MR analysis further strengthens the rationale for interventions targeting TyG‑associated metabolic pathways. Based on such stratification, preventive strategies should shift from a uniform approach to individualized management. Women with normal TyG profiles may benefit primarily from standard lifestyle counseling, whereas those with elevated indices, indicating ongoing metabolic impairment, should be prioritized for more intensive, targeted interventions. These may include structured, evidence-based programs focusing on diet, physical activity, and weight management to enhance insulin sensitivity, combined with closer monitoring of blood pressure, glucose, and lipid levels.

### Personalized medicine

The core principle of personalized medicine is to translate biomarker discoveries into robust, actionable tools for clinical decision-making. Personalized care plans should be tailored to each woman’s TyG profile and broader risk context. For women with elevated TyG indices, especially those who are young, normotensive, or non-obese, clinicians should adopt a lower threshold for enhanced follow-up and earlier initiation of lifestyle or pharmacologic measures aimed at reducing insulin resistance and visceral adiposity. In this way, TyG-guided management transforms HDP from a purely historical risk factor into an actionable entry point for long-term cardiovascular health optimization. This evidence-based approach supports a shift in HDP management from empirical, one-size-fits-all strategies to personalized medicine, establishing a solid foundation for lifelong cardiovascular care in women with HDP and promoting the transition from reactive to predictive medical practice.

### Contribution to the paradigm shift

Effectively identifying modifiable metabolic risk factors offers opportunities for implementing PPPM strategies in cardiovascular prevention for women with a history of HDP, thereby contributing to the extension of healthy lifespan. By applying the TyG indices, a simple and cost-effective biomarker, we have achieved three key shifts: quantitative risk prediction across a wide range of subclinical stages; targeted prevention during the critical postpartum period for women with ongoing metabolic vulnerability; and the design of individualized care plans based on each patient’s unique biological status rather than medical history alone. This approach promotes a transition from treating diseases after their onset to proactive prevention early in the postpartum period.

The TyG index provides a practical tool for implementing PPPM on a large scale among millions of women with a history of HDP. By transforming retrospective obstetric diagnoses into proactive, lifelong cardiovascular health management strategies, our study reframes HDP from a clinical challenge into an opportunity for early intervention and individualized prevention. Effective PPPM strategies underscore the immense potential and public health significance of metabolically informed cardiovascular disease prevention.

## Supplementary Information

Below is the link to the electronic supplementary material.

Supplementary file1 (DOCX 24 KB)

Supplementary file2 (DOCX 22 KB)

Supplementary file3 (PDF 68 KB)

Supplementary file4 (DOCX 28 KB)

Supplementary file5 (DOCX 22 KB)

Supplementary file6 Linear Relationship between TyG indices and postpartum cardiovascular disease risk. Abbreviations: CI, confidence interval; PCVD, postpartum cardiovascular disease; HR, hazard ratio; TyG, triglyceride-glucose; TyG-BMI, TyG-body mass index; TyG-WC, TyG-waist circumference; TyG-WHtR, TyG-waist-to-height ratio. Note: Restricted cubic spline analysis depicting the association between the TyG index (A) and modified TyG indices (B-D) with the incidence of cardiovascular disease among women with a history of hypertensive disorders of pregnancy. Models for panels B-D were adjusted for age at enrolment, age at first live birth, race, education levels, current smoking, current drinking, obesity, multiple live birth, history of hypertension, diabetes and chronic kidney disease. *Model for panel A was adjusted for age at enrolment, age at first live birth, race, education levels, current smoking, current drinking, obesity, multiple live birth, chronic kidney disease only. (TIF 173 KB)

Supplementary figure 1 (PNG 450 KB)

Supplementary file7 Comparative predictive performance of TyG indices for postpartum cardiovascular disease. Abbreviations: BMI, body mass index; PCVD, postpartum cardiovascular disease; TyG, triglyceride-glucose; WHtR, waist-to-height ratio; WC, waist circumference. Note: Time-dependent Harrell's C-indices for cardiovascular disease prediction using TyG index and its modified indices among women with prior hypertensive disorders of pregnancy. (TIF 327 KB)

Supplementary figure 2 (PNG 66.8 KB)

Supplementary file8 The scatter plot for the Mendelian randomization analyses of causal associations. (TIFF 5876 KB)

Supplementary figure 3 (PNG 1.79 MB)

Supplementary file9 Leave-one-out sensitivity analysis (TIFF 2455 KB)

Supplementary figure 4 (PNG 1.63 MB)

Supplementary file10 (TIFF 2408 KB)

Supplementary figure 5 (PNG 1.82 MB)

## Data Availability

The datasets generated and/or analyzed during the current study are available from the corresponding author upon reasonable request.

## Abbreviations

BMI: Body Mass Index
BWMR: Bayesian weighted Mendelian randomization
CI: Confidence Interval
CKD: Chronic Kidney Disease
CKM: Cardiovascular-Kidney-Metabolic
CVD: Cardiovascular Disease
DBP: Diastolic Blood Pressure
DM: Diabetes Mellitus
eGFR: Estimated Glomerular Filtration Rate
eNOS: Endothelial Nitric Oxide Synthase
FPG: Fasting Plasma Glucose
HbA1c: Glycated Hemoglobin A1c
HDL-C: High-Density Lipoprotein Cholesterol
HDP: Hypertensive Disorders of Pregnancy
HR: Hazard Ratio
ICD: International Classification of Diseases
IDI: Integrated Discrimination Improvement
IPAQ: International Physical Activity Questionnaire
IR: Insulin Resistance
IQR: Interquartile Range
LDL-C: Low-Density Lipoprotein Cholesterol
MR: Mendelian Randomization
NRI: Net Reclassification Improvement
PPPM: Predictive, preventive, and personalized medicine
RCS: Restricted Cubic Spline
ROC: Receiver Operating Characteristic
SBP: Systolic Blood Pressure
SD: Standard Deviation
STROBE: Strengthening the Reporting of Observational Studies in Epidemiology
TG: Triglycerides
TyG: Triglyceride-Glucose index
TyG-BMI: Triglyceride-Glucose index–Body Mass Index
TyG-WC: Triglyceride-Glucose index-Waist Circumference
TyG-WHtR: Triglyceride-Glucose index-Waist-to-Height Ratio
