# Supplementary material for: Triglyceride glucose index and modified triglyceride glucose indices are instrumental to optimize 3P medical management for postpartum cardiovascular disease
Source: EPMA J. 2026 Feb 19;17(1):105–20. doi: 10.1007/s13167-026-00437-8 (PMC12976339; doi:10.1007/s13167-026-00437-8)
Supplement: Supplementary file 1 — Supplementary file1 (DOCX 24 KB) [file 13167_2026_437_MOESM1_ESM.docx]

**Supplementary**

Table S1. Sensitivity analyses of the relationship between TyG index and modified indices with postpartum CVD incidence

|  | TyG | | TyG-BMI | | TyG-WHtR | | TyG-WC | |
| --- | --- | --- | --- | --- | --- | --- | --- | --- |
|  | HR (95% CI) | *P*-value | HR (95% CI) | *P*-value | HR (95% CI) | *P*-value | HR (95% CI) | *P*-value |
| Sensitivity Analysis 1 |  |  |  |  |  |  |  |  |
| Continuous | 1.30 (1.04, 1.62) | 0.020^*^ | 1.01 (1.00, 1.02) | 0.004^**^ | 1.01 (1.00, 1.01) | 0.003^**^ | 1.00 (1.00, 1.01) | 0.003^**^ |
| Tertile |  |  |  |  |  |  |  |  |
| Low | ref |  | ref |  | ref |  | ref |  |
| Middle | 1.28 (0.98, 1.66) | 0.071 | 1.16 (0.89, 1.52) | 0.271 | 1.18 (0.90, 1.54) | 0.229 | 1.10 (0.84, 1.43) | 0.5044 |
| High | 1.40 (1.06, 1.86) | 0.019^*^ | 1.41 (1.05, 1.89) | 0.023^*^ | 1.37 (1.03, 1.84) | 0.032^*^ | 1.38 (1.04, 1.84) | 0.0278^*^ |
| Sensitivity Analysis 2 |  |  |  |  |  |  |  |  |
| Continuous | 1.34 (1.06, 1.70) | 0.014^*^ | 1.01 (1.00, 1.02) | 0.004^**^ | 1.01 (1.00, 1.01) | 0.002^**^ | 1.00 (1.00, 1.01) | 0.002^**^ |
| Tertile |  |  |  |  |  |  |  |  |
| Low | ref |  | ref |  | ref |  | ref |  |
| Middle | 1.36 (1.02, 1.80) | 0.036^*^ | 1.18 (0.88, 1.58) | 0.258 | 1.23 (0.93, 1.64) | 0.151 | 1.17 (0.88, 1.56) | 0.269 |
| High | 1.54 (1.14, 2.08) | 0.005^**^ | 1.52 (1.11, 2.09) | 0.009^**^ | 1.44 (1.06, 1.96) | 0.022^*^ | 1.45 (1.07, 1.97) | 0.018^**^ |

Abbreviations: BMI, body mass index; CI, conﬁdence interval; CVD: cardiovascular disease; HDP, hypertensive disorders of pregnancy; HR, hazard ratio; TyG, Triglyceride-glucose index; WHtR: Waist-to-height ratio; WC: Waist circumference.

Sensitivity analysis 1 adjusted for age at enrolment, age at first live birth, race, education levels, current smoking, current drinking, obesity, multiple live birth, history of hypertension, diabetes and chronic kidney disease, LDL, EGFR, and HbA1c; sensitivity analysis 2 excluded participants who followed up only to the first 2 years, remaining 1,586 participants.* *P*<0.05, ** *P*<0.01, *** *P*<0.001.
