# Supplementary material for: Triglyceride glucose index and modified triglyceride glucose indices are instrumental to optimize 3P medical management for postpartum cardiovascular disease
Source: EPMA J. 2026 Feb 19;17(1):105–20. doi: 10.1007/s13167-026-00437-8 (PMC12976339; doi:10.1007/s13167-026-00437-8)
Supplement: Supplementary file 2 — Supplementary file2 (DOCX 22 KB) [file 13167_2026_437_MOESM2_ESM.docx]

Table S2. NRI and IDI comparison among TyG and its modified indices.

| **Comparison** | **IDI (95% CI)** | ***P* value** |
| --- | --- | --- |
| TyG-BMI vs TyG index | 0.004 (-0.002, 0.009) | 0.219 |
| TyG-WHtR vs TyG index | 0.005 (0.001, 0.010) | 0.013^*^ |
| TyG-WC vs TyG index | 0.005 (0.001, 0.009) | 0.02^*^ |
| TyG-BMI vs TyG-WHtR | 0.001 (-0.001, 0.005) | 0.339 |
| TyG-BMI vs TyG-WC | 0.001 (-0.002, 0.005) | 0.458 |
| TyG-WHtR vs TyG-WC | 0.000 (-0.002, 0.002) | 0.658 |
| **Comparison** | **NRI (95% CI)** | ***P* value** |
| TyG-BMI vs TyG index | 0.056 (-0.066, 0.165) | 0.458 |
| TyG-WHtR vs TyG index | 0.072 (-0.025, 0.144) | 0.14 |
| TyG-WC vs TyG index | 0.077 (-0.026, 0.167) | 0.186 |
| TyG-BMI vs TyG-WHtR | 0.026 (-0.099, 0.126) | 0.618 |
| TyG-BMI vs TyG-WC | 0.044 (-0.103, 0.158) | 0.465 |
| TyG-WHtR vs TyG-WC | -0.064 (-0.139, 0.067) | 0.286 |

Abbreviations: BMI, body mass index; CI, conﬁdence interval; IDI, integrated discrimination index; NRI, net reclassification improvement; TyG, Triglyceride-glucose index; WHtR: Waist-to-height ratio; WC: Waist circumference.

Note: Negative lower bounds of the confidence intervals for IDI and NRI values indicate statistical uncertainty in discriminatory improvement estimations. This is a common phenomenon when comparing risk prediction models with modest differences in performance. Confidence intervals crossing zero suggest non-significant improvements in risk prediction. * *P*<0.05.
