# Supplementary material for: Triglyceride glucose index and modified triglyceride glucose indices are instrumental to optimize 3P medical management for postpartum cardiovascular disease
Source: EPMA J. 2026 Feb 19;17(1):105–20. doi: 10.1007/s13167-026-00437-8 (PMC12976339; doi:10.1007/s13167-026-00437-8)
Supplement: Supplementary file 3 — Supplementary file3 (PDF 68 KB) [file 13167_2026_437_MOESM3_ESM.pdf]

exposure	pval	se	beta	SNP	effect_allele	other_allele	eaf	F_statistic	R_squared
TyG	3.85e-10	0.00142769	-0.00893744	rs10206462	C	T	NA	39.18848221	0.975117249
TyG	2.96e-22	0.00154174	0.0149589	rs10260148	T	C	NA	94.14061638	0.989489242
TyG	6.75e-13	0.00153309	0.0110148	rs1035941	A	G	NA	51.61999167	0.980995816
TyG	9.04e-10	0.00147493	0.00903509	rs10783828	A	G	NA	37.52512499	0.974042914
TyG	2.06e-21	0.00137832	0.0130977	rs10786069	C	T	NA	90.30045978	0.989047153
TyG	4.53e-08	0.0018054	-0.00987338	rs10811661	C	T	NA	29.90782481	0.967645734
TyG	1.37e-16	0.00148245	-0.0122569	rs10832027	G	A	NA	68.35986193	0.98558244
TyG	5.35e-10	0.00150663	0.00935439	rs10861679	C	T	NA	38.5494089	0.974715172
TyG	1.34e-162	0.00143046	-0.0388967	rs10889332	T	C	NA	739.3908611	0.998649362
TyG	2.38e-09	0.00184368	-0.0110061	rs11006681	A	G	NA	35.63656368	0.972704864
TyG	1.91e-11	0.00173721	-0.0116623	rs11045171	G	A	NA	45.06753209	0.978292738
TyG	5.65e-21	0.00150196	-0.0141141	rs11075253	A	C	NA	88.30588488	0.98880253
TyG	8.09e-11	0.00138913	-0.00902819	rs11118610	C	A	NA	42.23919177	0.976872833
TyG	1.15e-32	0.00142319	-0.0169417	rs11134475	A	G	NA	141.7059895	0.992992586
TyG	1e-08	0.00211397	0.0121133	rs111585158	T	C	NA	32.83426558	0.970444164
TyG	4.59e-09	0.00310342	-0.0181908	rs11216122	T	G	NA	34.35757386	0.971717516
TyG	2.21e-12	0.00342403	0.0240392	rs11216236	T	C	NA	49.29069016	0.980115604
TyG	2.76e-14	0.00195835	-0.0149023	rs112740904	G	T	NA	57.90632177	0.983023893
TyG	1.7e-12	0.00301795	0.0212993	rs113344423	A	G	NA	49.80885755	0.980318392
TyG	1.07e-21	0.00465259	0.0445298	rs114165349	C	G	NA	91.60346957	0.989201269
TyG	3.46e-08	0.00487631	0.026901	rs115128825	A	C	NA	30.43366353	0.968186973
TyG	6.29e-15	0.00147145	-0.011475	rs11558471	G	A	NA	60.81551234	0.983822831
TyG	2.34e-47	0.00254055	-0.03673	rs11600380	C	T	NA	209.0192878	0.995238533
TyG	4.65e-14	0.00154515	0.0116533	rs11636087	C	T	NA	56.87960365	0.982722757
TyG	2.14e-10	0.00293615	0.0186479	rs11651957	A	G	NA	40.33698055	0.975808586
TyG	1.41e-08	0.00138378	-0.00784868	rs11657238	A	G	NA	32.17059896	0.969852821
TyG	8.79e-109	0.00491157	-0.108878	rs116843064	A	G	NA	491.4050251	0.997969152
TyG	0	0.00226332	-0.0951759	rs117026536	T	G	NA	1768.3249	0.999434813
TyG	2.48e-13	0.00439412	-0.0321654	rs117805502	T	C	NA	53.5838713	0.98167957
TyG	1.85e-08	0.00176553	0.00993171	rs11781356	A	T	NA	31.64447223	0.969366942
TyG	4.07e-08	0.00176945	0.00971075	rs12173130	C	T	NA	30.11819611	0.967864461
TyG	7.75e-16	0.00272455	0.0219558	rs12208357	T	C	NA	64.93950403	0.984834584
TyG	4.76e-37	0.00147498	-0.0187602	rs12446515	T	C	NA	161.7717431	0.993856428
TyG	2.24e-14	0.00183258	0.013994	rs12610709	A	G	NA	58.31198883	0.983140002
TyG	1.09e-12	0.00200585	0.0142794	rs12617848	T	C	NA	50.67841378	0.980649561
TyG	2.46e-16	0.0039376	-0.0322797	rs12721078	A	C	NA	67.20410272	0.985338125
TyG	2.11e-08	0.00162151	0.00908515	rs12885801	A	C	NA	31.39243051	0.96912859
TyG	5.15e-12	0.00195818	0.0135148	rs12934528	C	T	NA	47.63366798	0.979438113
TyG	8.64e-09	0.00189096	0.0108836	rs12937081	G	A	NA	33.12687428	0.97069758
TyG	3.86e-08	0.00218488	0.0120107	rs13074711	C	T	NA	30.21909544	0.967968322
TyG	1.54e-27	0.00143815	0.0156395	rs13108218	A	G	NA	118.2598592	0.991614949
TyG	1.37e-12	0.00166888	-0.0118284	rs13289566	T	C	NA	50.23446543	0.980481889
TyG	8.01e-23	0.00145941	0.0143536	rs1388941	A	G	NA	96.73130417	0.989767864
TyG	5.34e-59	0.00443008	0.0717689	rs139974673	C	T	NA	262.4517312	0.996204238
TyG	1.71e-10	0.00278129	-0.0177614	rs142164605	A	T	NA	40.78137283	0.976065889
TyG	2.05e-08	0.00307793	0.0172612	rs144470864	C	A	NA	31.45027824	0.969183623
TyG	3.22e-16	0.00435052	0.0355235	rs146390218	G	A	NA	66.67287125	0.98522303
TyG	1.56e-31	0.00140761	0.0164471	rs1471251	T	A	NA	136.5255639	0.992728625
TyG	4.18e-08	0.00550582	-0.03019	rs147764624	C	G	NA	30.06645379	0.967810938
TyG	2.6e-08	0.00141384	0.00786984	rs151913	A	G	NA	30.98355717	0.968733934
TyG	2.64e-37	0.0014104	0.0180035	rs1532085	A	G	NA	162.9405911	0.993900229
TyG	6.18e-15	0.00137638	0.0107368	rs1534696	C	A	NA	60.85174267	0.983832307
TyG	4.64e-09	0.001496	0.00876614	rs1585705	C	A	NA	34.3363097	0.971700497
TyG	6.14e-12	0.00252122	-0.0173377	rs16836630	C	G	NA	47.28914705	0.979291413
TyG	1.82e-75	0.00427426	0.0785949	rs17091881	C	T	NA	338.1168845	0.997051164
TyG	2.91e-13	0.00285324	0.0208253	rs17092008	T	C	NA	53.27284923	0.981574581
TyG	4.94e-23	0.00375036	0.0370675	rs17119701	G	A	NA	97.68787814	0.989867043
TyG	5.28e-200	0.00185642	-0.0560614	rs17145750	T	C	NA	911.959183	0.998904661
TyG	4.9e-27	0.00139861	-0.0150613	rs1716407	G	A	NA	115.9662624	0.991450526
TyG	2.01e-224	0.00137141	-0.0439	rs17321515	G	A	NA	1024.694527	0.999025051
TyG	3.19e-09	0.0015125	-0.00895698	rs1760801	A	G	NA	35.06973162	0.972275923
TyG	2.65e-09	0.00147002	0.00874937	rs17656269	T	C	NA	35.4247732	0.972546157
TyG	2.19e-10	0.00141859	0.00900471	rs17694506	C	T	NA	40.29263534	0.975782606
TyG	6.78e-13	0.00408097	-0.0293183	rs1801689	C	A	NA	51.61200902	0.980992933
TyG	8.63e-10	0.00338431	0.0207564	rs185139895	A	G	NA	37.61523579	0.974103486
TyG	1.51e-15	0.00592501	-0.0472632	rs186696265	T	C	NA	63.63090057	0.984527525

TyG	8.06e-09	0.00540272	0.031159	rs187217942	A	G	NA	33.26152073	0.970812737
TyG	1.07e-36	0.00606752	-0.0767868	rs187929675	T	C	NA	160.1587628	0.993794939
TyG	1.02e-23	0.00642107	-0.0644712	rs188247550	T	C	NA	100.8130371	0.990178075
TyG	8.04e-20	0.00148108	-0.0134975	rs1882491	C	T	NA	83.05191245	0.988102591
TyG	2.25e-25	0.0013714	-0.0142769	rs1967685	C	G	NA	108.3776039	0.990857361
TyG	1.99e-10	0.00145381	-0.00925016	rs197156	G	A	NA	40.48397854	0.975894308
TyG	1.54e-10	0.00249077	-0.0159447	rs2035816	G	A	NA	40.9793865	0.976178785
TyG	2.19e-11	0.00579905	0.0388118	rs2075294	T	G	NA	44.79338293	0.978162784
TyG	9.63e-16	0.00145382	0.011677	rs2081687	T	C	NA	64.51211529	0.984735648
TyG	3.59e-14	0.00142823	-0.0108194	rs2106727	A	G	NA	57.38654151	0.982872765
TyG	4.17e-08	0.00146386	-0.00802754	rs213498	A	T	NA	30.07224624	0.967816939
TyG	2.83e-13	0.00387832	0.0283225	rs2207132	A	G	NA	53.33052896	0.981594142
TyG	2.62e-10	0.00211876	-0.0133902	rs2244278	A	C	NA	39.94023327	0.97557415
TyG	4.1e-08	0.00163964	0.00899617	rs2250900	T	C	NA	30.10359053	0.967849371
TyG	5.43e-11	0.00138503	-0.00908433	rs2277844	G	A	NA	43.01970654	0.977282902
TyG	4.71e-08	0.00162296	0.00886478	rs2302883	C	T	NA	29.83460496	0.967568905
TyG	3.76e-12	0.00188077	0.0130645	rs231539	T	C	NA	48.25187664	0.979696205
TyG	9.88e-55	0.00167767	0.0261448	rs261334	G	C	NA	242.8608836	0.995899301
TyG	1.25e-98	0.00515243	0.10865	rs268	G	A	NA	444.667369	0.997756174
TyG	1.76e-09	0.00137239	0.00826032	rs2745400	A	G	NA	36.22752411	0.973138155
TyG	6.4e-11	0.00153811	-0.0100505	rs2792736	T	A	NA	42.69731292	0.977115298
TyG	2.42e-22	0.00182104	-0.0177064	rs28550053	G	A	NA	94.54130031	0.989533322
TyG	1.46e-15	0.00218851	0.0174652	rs2894211	A	C	NA	63.68689207	0.984540918
TyG	6.79e-25	0.00149811	0.0154375	rs2925979	T	C	NA	106.1858225	0.990670408
TyG	2.4e-48	0.0014316	-0.0209209	rs2943645	C	T	NA	213.558739	0.995339272
TyG	3.58e-15	0.00169886	0.0133691	rs2963476	G	A	NA	61.92830049	0.984108899
TyG	2.61e-08	0.00239848	0.0133494	rs2971676	A	G	NA	30.97785125	0.968728355
TyG	1.53e-09	0.0017554	0.0106046	rs2975424	C	T	NA	36.49525442	0.973329958
TyG	2.4e-10	0.00212481	-0.0134581	rs3025053	A	G	NA	40.1168938	0.975679097
TyG	4.45e-11	0.0018033	0.0118809	rs3120619	A	G	NA	43.40729427	0.977481177
TyG	4.71e-10	0.00139646	-0.00869811	rs340836	C	T	NA	38.79652169	0.974872176
TyG	2.97e-08	0.00285011	-0.0157999	rs34820917	A	G	NA	30.73161846	0.968485692
TyG	6.21e-09	0.00144997	0.00842606	rs34921778	G	A	NA	33.77000004	0.971239574
TyG	2.61e-18	0.00283283	0.0247241	rs35169799	T	C	NA	76.1728065	0.987042068
TyG	5.32e-10	0.00149721	0.00929684	rs35477346	C	T	NA	38.55718109	0.97472014
TyG	3.09e-15	0.00234709	0.0185128	rs35750610	C	T	NA	62.21351403	0.984180598
TyG	6.27e-11	0.00189812	-0.0124087	rs3750571	A	C	NA	42.73711873	0.977136125
TyG	7.84e-09	0.00170108	0.00981886	rs3808976	G	A	NA	33.31751934	0.970860365
TyG	3.87e-08	0.00143983	0.00791466	rs38205	A	C	NA	30.21635608	0.967965511
TyG	3.5e-10	0.0013818	0.00867089	rs3822076	A	T	NA	39.37648783	0.975233111
TyG	1.73e-09	0.00220378	-0.0132704	rs390556	C	T	NA	36.26031333	0.973161793
TyG	3.63e-18	0.00176462	-0.0153354	rs390802	A	G	NA	75.52449368	0.986932289
TyG	3.69e-35	0.00174659	0.0216126	rs3936511	G	A	NA	153.1200608	0.993511552
TyG	1.86e-08	0.00588779	-0.0331177	rs41290102	T	C	NA	31.6384874	0.969361325
TyG	2.37e-08	0.00170166	-0.00950005	rs4134363	A	G	NA	31.16780382	0.968913016
TyG	3.39e-18	0.00149941	-0.0130424	rs419925	C	G	NA	75.66137425	0.986955621
TyG	3.11e-08	0.00203182	-0.0112466	rs4709746	T	C	NA	30.63882009	0.968393259
TyG	1.3e-11	0.00143947	0.00974329	rs4715317	T	G	NA	45.81482393	0.978639245
TyG	5.21e-23	0.00188091	-0.0185803	rs4722551	C	T	NA	97.58193214	0.989856153
TyG	7.53e-12	0.00138516	0.00948499	rs4804413	T	C	NA	46.88931317	0.979118514
TyG	1.64e-08	0.00161739	0.0091331	rs4812995	C	T	NA	31.88650489	0.96959239
TyG	8.09e-168	0.00161686	0.0446753	rs483082	T	G	NA	763.4667382	0.998691899
TyG	3.31e-29	0.00141892	-0.0159198	rs484066	A	T	NA	125.880771	0.992118585
TyG	8.63e-52	0.00146286	0.0221545	rs4846922	T	C	NA	229.3603677	0.995658976
TyG	1.07e-31	0.00165914	0.0194386	rs4921914	C	T	NA	137.2662909	0.992767579
TyG	8.59e-14	0.00248476	-0.0185398	rs5110	A	C	NA	55.67256044	0.982354776
TyG	6.48e-41	0.00346743	-0.0464535	rs533617	C	T	NA	179.4822385	0.994459289
TyG	6.94e-12	0.00353606	0.0242543	rs541012177	T	G	NA	47.04768287	0.979187342
TyG	2.05e-22	0.00360432	0.0351072	rs55697600	G	A	NA	94.87367057	0.989569608
TyG	8.74e-13	0.0025524	-0.0182481	rs55730499	T	C	NA	51.11376036	0.98081121
TyG	0	0.00278601	0.108415	rs56225305	A	G	NA	1514.305175	0.999340067
TyG	4.65e-11	0.00302387	-0.019903	rs57192995	C	G	NA	43.32223494	0.977437961
TyG	8.6e-11	0.00469748	-0.0304863	rs57295072	C	G	NA	42.11914615	0.976808446
TyG	1.26e-90	0.00257716	-0.0520456	rs58542926	T	C	NA	407.8357337	0.99755403
TyG	1.18e-13	0.00298074	0.0221143	rs5880	C	G	NA	55.04250652	0.982156401
TyG	1.75e-12	0.00180395	0.0127238	rs58895965	A	C	NA	49.74903671	0.980295192
TyG	2.36e-08	0.00152344	-0.00850564	rs6066138	A	G	NA	31.17190082	0.968916975
TyG	7.14e-57	0.00172428	0.0274094	rs6073958	C	T	NA	252.6873355	0.99605814

TyG	1.33e-10	0.00138822	0.0089174	rs6090040	A	C	NA	41.26291467	0.976338594
TyG	1.06e-22	0.00142207	-0.0139461	rs61362984	G	A	NA	96.17531129	0.98970932
TyG	1.57e-22	0.00292878	-0.028607	rs61737373	A	G	NA	95.40499906	0.989627094
TyG	1.15e-08	0.00409645	0.0233784	rs61904855	A	C	NA	32.56973713	0.970211265
TyG	3.22e-14	0.00152691	0.0115887	rs62102718	T	A	NA	57.60266304	0.982935929
TyG	1.29e-09	0.00150245	-0.00911832	rs62132802	T	C	NA	36.83236474	0.973567605
TyG	7.15e-17	0.00303878	0.0253586	rs62271373	A	T	NA	69.6389201	0.985843498
TyG	4.32e-09	0.00364332	-0.0213924	rs62459110	C	G	NA	34.47659644	0.971812403
TyG	5.11e-21	0.00155763	0.0146537	rs62521590	G	T	NA	88.50462903	0.988827394
TyG	2.94e-27	0.00142077	0.0153665	rs632057	T	G	NA	116.9775077	0.991523808
TyG	1.95e-08	0.00149284	0.00838469	rs6437249	T	C	NA	31.5462321	0.969274477
TyG	1.49e-11	0.00187808	0.0126753	rs6448429	T	C	NA	45.5499934	0.97851772
TyG	2.05e-161	0.00138448	0.0375073	rs6547692	G	A	NA	733.9360692	0.998639337
TyG	2.76e-08	0.00161797	-0.00898973	rs6710938	C	A	NA	30.87111865	0.96862363
TyG	1.55e-08	0.00137713	-0.00778921	rs6760053	G	C	NA	31.99167263	0.969689321
TyG	1.04e-09	0.00153217	0.00935072	rs678614	A	C	NA	37.24570473	0.973853273
TyG	8.09e-13	0.00207488	0.0148562	rs67981690	G	A	NA	51.26600858	0.980867106
TyG	1.67e-12	0.00154721	-0.0109234	rs6923241	T	C	NA	49.84448889	0.980332185
TyG	2.5e-09	0.00273609	-0.0163114	rs71352934	C	A	NA	35.54035146	0.972632994
TyG	1.61e-21	0.00150803	0.0143695	rs7140110	C	T	NA	90.79529644	0.989106196
TyG	2.24e-20	0.00211444	-0.0195603	rs71480323	A	G	NA	85.57763706	0.988449673
TyG	1.42e-14	0.00254745	0.019603	rs71525127	G	C	NA	59.21527391	0.983392918
TyG	1.15e-09	0.00205483	0.0125089	rs71603401	G	A	NA	37.05838133	0.973724579
TyG	9.44e-09	0.00141367	-0.00811532	rs7175132	G	A	NA	32.95453707	0.970548855
TyG	6.53e-09	0.00320686	0.018609	rs72669514	T	C	NA	33.67333032	0.97115939
TyG	4.04e-09	0.00206079	-0.0121232	rs72739147	T	A	NA	34.60724785	0.97191583
TyG	9.58e-12	0.00314107	-0.0213999	rs72754154	A	G	NA	46.41605841	0.978910099
TyG	4.88e-10	0.00235286	-0.0146422	rs72801474	A	G	NA	38.72759217	0.974828578
TyG	1.69e-68	0.00389919	0.0682178	rs72836561	T	C	NA	306.0882249	0.996743607
TyG	1.2e-08	0.00240632	-0.0137147	rs72904790	C	T	NA	32.48372782	0.970134747
TyG	4.84e-08	0.00217251	-0.0118561	rs7296326	C	T	NA	29.78243064	0.967513936
TyG	3.68e-08	0.00222876	0.0122709	rs73198299	C	T	NA	30.31281188	0.96806419
TyG	8.82e-13	0.00488632	0.034928	rs74444445	C	T	NA	51.09561451	0.980804526
TyG	1.3e-10	0.00434519	-0.0279294	rs75662196	C	G	NA	41.31480137	0.976367607
TyG	2.56e-49	0.0031699	-0.046805	rs75919952	T	C	NA	218.0185141	0.995434176
TyG	2.64e-09	0.00383242	0.0228139	rs76172548	C	A	NA	35.43666731	0.972555119
TyG	1.16e-31	0.0025237	-0.0295509	rs76384951	C	A	NA	137.1090018	0.992759342
TyG	5.86e-18	0.00259593	0.0224178	rs77009508	G	A	NA	74.57616145	0.986768314
TyG	1.84e-12	0.00155172	-0.0109345	rs7703744	G	C	NA	49.65593254	0.980258976
TyG	4.09e-09	0.00148835	-0.00875261	rs77244849	C	T	NA	34.58318688	0.97189684
TyG	4.52e-18	0.00164795	0.0142803	rs7758790	C	T	NA	75.09077746	0.986857803
TyG	1.96e-08	0.00482777	0.0271124	rs78025076	T	C	NA	31.53862701	0.969267296
TyG	6.39e-22	0.00169741	0.0163357	rs7821812	C	G	NA	92.61940473	0.989318454
TyG	0	0.00278666	0.124688	rs7930786	C	G	NA	2002.079645	0.999500769
TyG	1.92e-09	0.0062985	0.0378212	rs79429216	A	G	NA	36.0575605	0.973014953
TyG	4.05e-33	0.00340184	0.0407906	rs799157	T	C	NA	143.7779941	0.993092873
TyG	2.49e-29	0.00210804	-0.0237048	rs79953491	G	A	NA	126.4488536	0.992153715
TyG	3.05e-09	0.00238957	-0.0141686	rs80216311	T	C	NA	35.15720691	0.972342996
TyG	1.13e-09	0.00158419	0.00964703	rs8025505	T	C	NA	37.08281744	0.973741439
TyG	6.53e-11	0.00137468	-0.00897866	rs8028620	C	T	NA	42.6598991	0.977095687
TyG	8.69e-09	0.00141115	0.00812063	rs8092347	G	A	NA	33.11563493	0.970687926
TyG	5.55e-09	0.0014628	0.00852818	rs852424	T	C	NA	33.98934638	0.971419872
TyG	8.43e-43	0.00158662	0.0217619	rs878521	A	G	NA	188.1255453	0.994712507
TyG	1.16e-22	0.00162585	0.0159306	rs904009	C	A	NA	96.00709203	0.989691475
TyG	2.31e-11	0.00138756	-0.00927654	rs907866	A	G	NA	44.69598368	0.978116239
TyG	4.54e-10	0.00159406	0.00993826	rs9891030	A	G	NA	38.86971717	0.974918307
TyG	2.49e-08	0.00177279	0.00988172	rs9935836	C	A	NA	31.07066162	0.968818853
TyG	5.73e-45	0.00143553	0.020203	rs99780	T	C	NA	198.0647462	0.994976509
