# Supplementary material for: Triglyceride glucose index and modified triglyceride glucose indices are instrumental to optimize 3P medical management for postpartum cardiovascular disease
Source: EPMA J. 2026 Feb 19;17(1):105–20. doi: 10.1007/s13167-026-00437-8 (PMC12976339; doi:10.1007/s13167-026-00437-8)
Supplement: Supplementary file 4 — Supplementary file4 (DOCX 28 KB) [file 13167_2026_437_MOESM4_ESM.docx]

| **Table S4. Summary of Original and FDR-Adjusted P-Values** | | | | | | | |
| --- | --- | --- | --- | --- | --- | --- | --- |
| **Id.outcome** | **Outcome** | **Exposure** | **Method** | **Nsnp** | **OR** | **Original P-Value** | **FDR-Adjusted P-Value** |
| finn-b-FG_CVD | CVD | TyG | MR Egger | 90 | 1.123 | 0.187589 | 0.559511 |
| finn-b-FG_CVD | CVD | TyG | Inverse variance weighted | 90 | 1.212 | 0.000319 | 0.000638 |
| finn-b-FG_CVD | CVD | TyG | Weighted mode | 90 | 1.192 | 0.015113 | 0.045339 |
| ebi-a-GCST90029019 | CVD | TyG | MR Egger | 89 | 1.066 | 6.70E-15 | 3.35E-14 |
| ebi-a-GCST90029019 | CVD | TyG | Inverse variance weighted | 89 | 1.145 | 1.36E-66 | 6.80E-66 |
| ebi-a-GCST90029019 | CVD | TyG | Weighted mode | 89 | 1.115 | 1.40E-22 | 7.00E-22 |
| finn-b-FG_CVD | CVD | BMI | MR Egger | 36 | 1.169 | 8.44E-06 | 4.22E-05 |
| finn-b-FG_CVD | CVD | BMI | Inverse variance weighted | 36 | 1.102 | 4.50E-17 | 2.25E-16 |
| finn-b-FG_CVD | CVD | BMI | Weighted mode | 36 | 1.132 | 1.75E-07 | 8.75E-07 |
| ebi-a-GCST90029019 | CVD | BMI | MR Egger | 36 | 1.026 | 3.23E-06 | 1.29E-05 |
| ebi-a-GCST90029019 | CVD | BMI | Inverse variance weighted | 36 | 1.019 | 1.93E-26 | 7.72E-26 |
| ebi-a-GCST90029019 | CVD | BMI | Weighted mode | 36 | 1.020 | 7.27E-08 | 1.45E-07 |
| finn-b-FG_CVD | CVD | WC | MR Egger | 40 | 2.012 | 2.89E-05 | 0.000116 |
| finn-b-FG_CVD | CVD | WC | Inverse variance weighted | 40 | 1.593 | 1.68E-16 | 6.72E-16 |
| finn-b-FG_CVD | CVD | WC | Weighted mode | 40 | 1.798 | 1.53E-06 | 6.12E-06 |
| ebi-a-GCST90029019 | CVD | WC | MR Egger | 41 | 1.137 | 0.002478 | 0.004956 |
| ebi-a-GCST90029019 | CVD | WC | Inverse variance weighted | 41 | 1.095 | 7.04E-10 | 1.41E-09 |
| ebi-a-GCST90029019 | CVD | WC | Weighted mode | 41 | 1.122 | 2.71E-14 | 1.08E-13 |
| finn-b-FG_CVD | CVD | WHR | MR Egger | 305 | 1.113 | 0.186504 | 0.559511 |
| finn-b-FG_CVD | CVD | WHR | Inverse variance weighted | 305 | 1.088 | 0.013817 | 0.013817 |
| finn-b-FG_CVD | CVD | WHR | Weighted mode | 305 | 1.070 | 0.32285 | 0.343824 |
| ebi-a-GCST90029019 | CVD | WHR | MR Egger | 310 | 1.075 | 0.000184 | 0.000553 |
| ebi-a-GCST90029019 | CVD | WHR | Inverse variance weighted | 310 | 1.065 | 5.20E-15 | 1.56E-14 |
| ebi-a-GCST90029019 | CVD | WHR | Weighted mode | 310 | 1.062 | 3.42E-08 | 1.03E-07 |
| finn-b-FG_CVD | CVD | HDP | MR Egger | 33 | 0.994 | 0.944562 | 0.944562 |
| finn-b-FG_CVD | CVD | HDP | Inverse variance weighted | 33 | 1.167 | 8.24E-06 | 2.47E-05 |
| finn-b-FG_CVD | CVD | HDP | Weighted mode | 33 | 1.071 | 0.171912 | 0.343824 |
| ebi-a-GCST90029019 | CVD | HDP | MR Egger | 31 | 0.983 | 0.481052 | 0.481052 |
| ebi-a-GCST90029019 | CVD | HDP | Inverse variance weighted | 31 | 1.063 | 0.000317 | 0.000317 |
| ebi-a-GCST90029019 | CVD | HDP | Weighted mode | 31 | 1.010 | 0.026566 | 0.026566 |
| finn-b-FG_CVD | CVD | TyG | BWMR | 90 | 1.238 | 0.000216 | 4.32E-04 |
| ebi-a-GCST90029019 | CVD | TyG | BWMR | 89 | 1.138 | 1.21E-26 | 1.21E-25 |
| finn-b-FG_CVD | CVD | BMI | BWMR | 36 | 1.101 | 1.33E-19 | 9.31E-19 |
| ebi-a-GCST90029019 | CVD | BMI | BWMR | 36 | 1.017 | 4.93E-21 | 4.44E-20 |
| finn-b-FG_CVD | CVD | WC | BWMR | 40 | 1.608 | 1.34E-16 | 8.04E-16 |
| ebi-a-GCST90029019 | CVD | WC | BWMR | 41 | 1.094 | 1.46E-09 | 5.84E-09 |
| finn-b-FG_CVD | CVD | WHR | BWMR | 305 | 1.096 | 0.007255 | 7.25E-03 |
| ebi-a-GCST90029019 | CVD | WHR | BWMR | 310 | 1.070 | 1.50E-20 | 1.20E-19 |
| finn-b-FG_CVD | CVD | HDP | BWMR | 33 | 1.637 | 7.29E-07 | 2.19E-06 |
| ebi-a-GCST90029019 | CVD | HDP | BWMR | 31 | 1.150 | 1.78E-13 | 8.90E-13 |

Abbreviations: BMI, body mass index; BWMR, Bayesian weighted Mendelian randomization; CVD, cardiovascular disease; FDR, false discovery rate; HDP, hypertensive disorders of pregnancy; MR, Mendelian randomization; OR, odds ratio; TyG, triglyceride-glucose; WHR, waist-to-hip ratio; WC, waist circumference.
