# Supplementary material for: Triglyceride glucose index and modified triglyceride glucose indices are instrumental to optimize 3P medical management for postpartum cardiovascular disease
Source: EPMA J. 2026 Feb 19;17(1):105–20. doi: 10.1007/s13167-026-00437-8 (PMC12976339; doi:10.1007/s13167-026-00437-8)
Supplement: Supplementary file 5 — Supplementary file5 (DOCX 22 KB) [file 13167_2026_437_MOESM5_ESM.docx]

**Table S5. Heterogeneity tests and MR-Egger intercept of MR.**

| **Exposure** | **Outcome** | **Outcome id** | **Egger Intercept** | **P value** | **Cochran’s Q** | **P value** |
| --- | --- | --- | --- | --- | --- | --- |
| TyG | CVD | finn-b-FG_CVD | 0.001 | 0.58 | 289.55 | ＜0.001^***^ |
| TyG | CVD | ebi-a-GCST90029019 | 0.002 | 0.02^*^ | 566.58 | ＜0.001^***^ |
| BMI | CVD | finn-b-FG_CVD | -0.010 | 0.04^*^ | 49.337 | 0.04^*^ |
| BMI | CVD | ebi-a-GCST90029019 | -0.001 | 0.12 | 91.46 | ＜0.001^***^ |
| WC | CVD | finn-b-FG_CVD | -0.007 | 0.10 | 53.93 | 0.05 |
| WC | CVD | ebi-a-GCST90029019 | -0.001 | 0.32 | 290.49 | ＜0.001^***^ |
| WHR | CVD | finn-b-FG_CVD | -0.001 | 0.76 | 530.82 | ＜0.001^***^ |
| WHR | CVD | ebi-a-GCST90029019 | -0.0002 | 0.57 | 2206.11 | ＜0.001^***^ |
| HDP | CVD | finn-b-FG_CVD | 0.021 | 0.06 | 126.61 | ＜0.001^***^ |
| HDP | CVD | ebi-a-GCST90029019 | 0.007 | 0.03^*^ | 630.16 | ＜0.001^***^ |

Abbreviations: BMI, body mass index; CVD, cardiovascular disease; HDP, hypertensive disorders of pregnancy; MR, Mendelian randomization; TyG, triglyceride-glucose; WHR, waist-to-hip ratio; WC, waist circumference. * *P*<0.05, ** *P*<0.01, *** *P*<0.001.
