# Supplementary figures and images for: Triglyceride glucose index and modified triglyceride glucose indices are instrumental to optimize 3P medical management for postpartum cardiovascular disease
Source: EPMA J. 2026 Feb 19;17(1):105–20. doi: 10.1007/s13167-026-00437-8 (PMC12976339; doi:10.1007/s13167-026-00437-8)

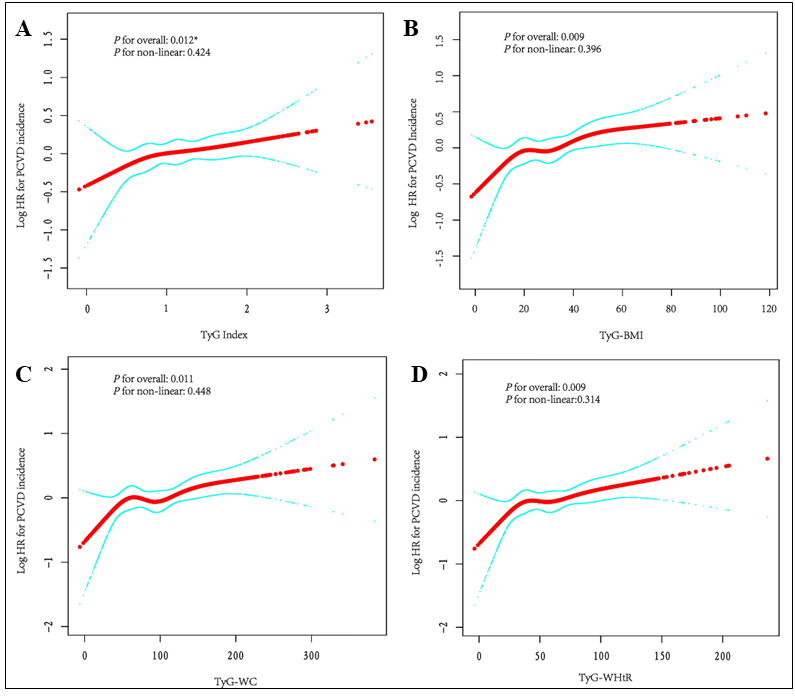

Supplement: Supplementary file 6 — Supplementary file6 Linear Relationship between TyG indices and postpartum cardiovascular disease risk. Abbreviations: CI, confidence interval; PCVD, postpartum cardiovascular disease; HR, hazard ratio; TyG, triglyceride-glucose; TyG-BMI, TyG-body mass index; TyG-WC, TyG-waist circumference; TyG-WHtR, TyG-waist-to-height ratio. Note: Restricted cubic spline analysis depicting the association between the TyG index (A) and modified TyG indices (B-D) with the incidence of cardiovascular disease among women with a history of hypertensive disorders of pregnancy. Models for panels B-D were adjusted for age at enrolment, age at first live birth, race, education levels, current smoking, current drinking, obesity, multiple live birth, history of hypertension, diabetes and chronic kidney disease. *Model for panel A was adjusted for age at enrolment, age at first live birth, race, education levels, current smoking, current drinking, obesity, multiple live birth, chronic kidney disease only. (TIF 173 KB) [file 13167_2026_437_MOESM6_ESM.tif]

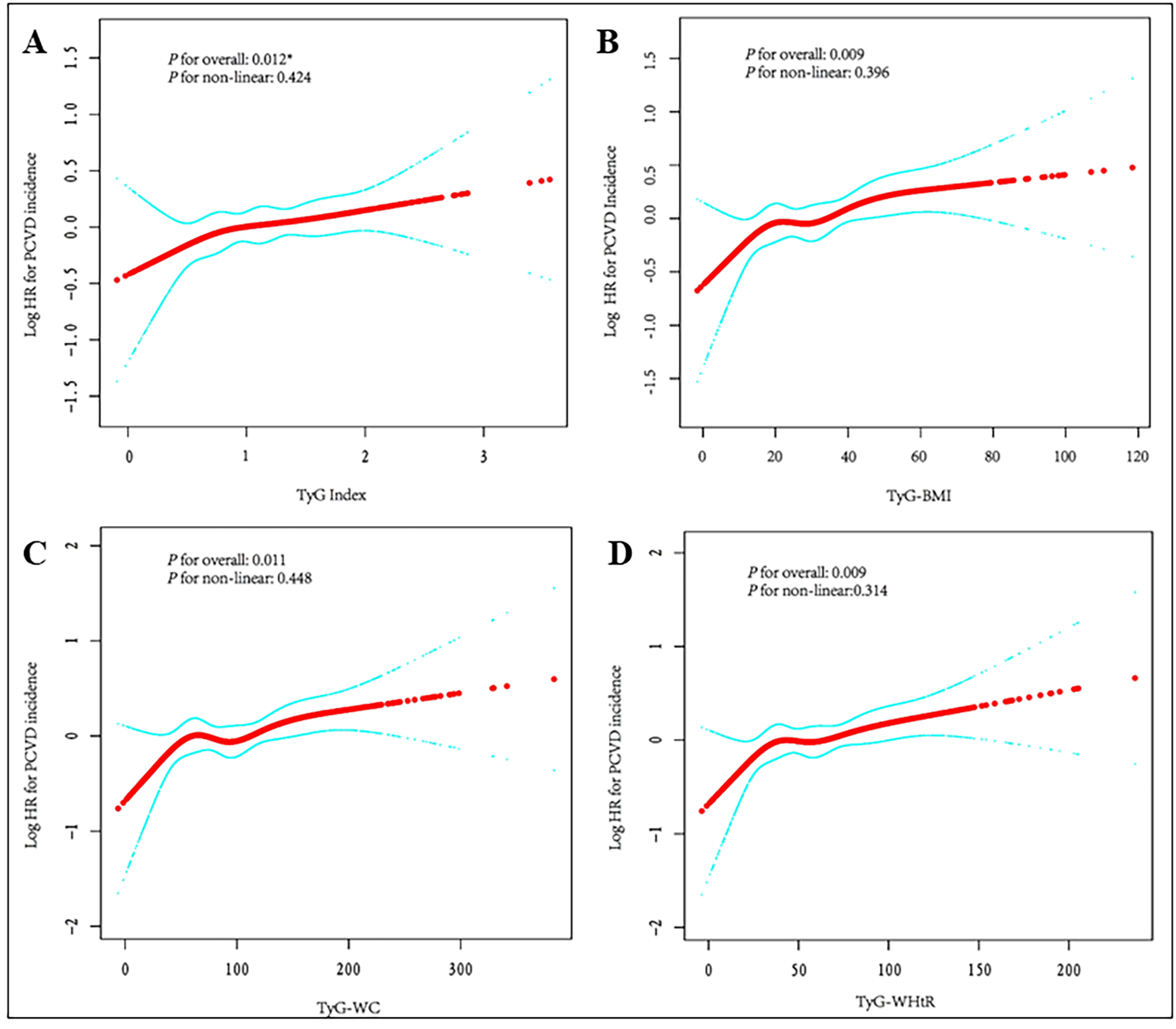

Supplement: Supplementary file 7 — (PNG 450 KB) [file 13167_2026_437_Fig4_ESM.png]

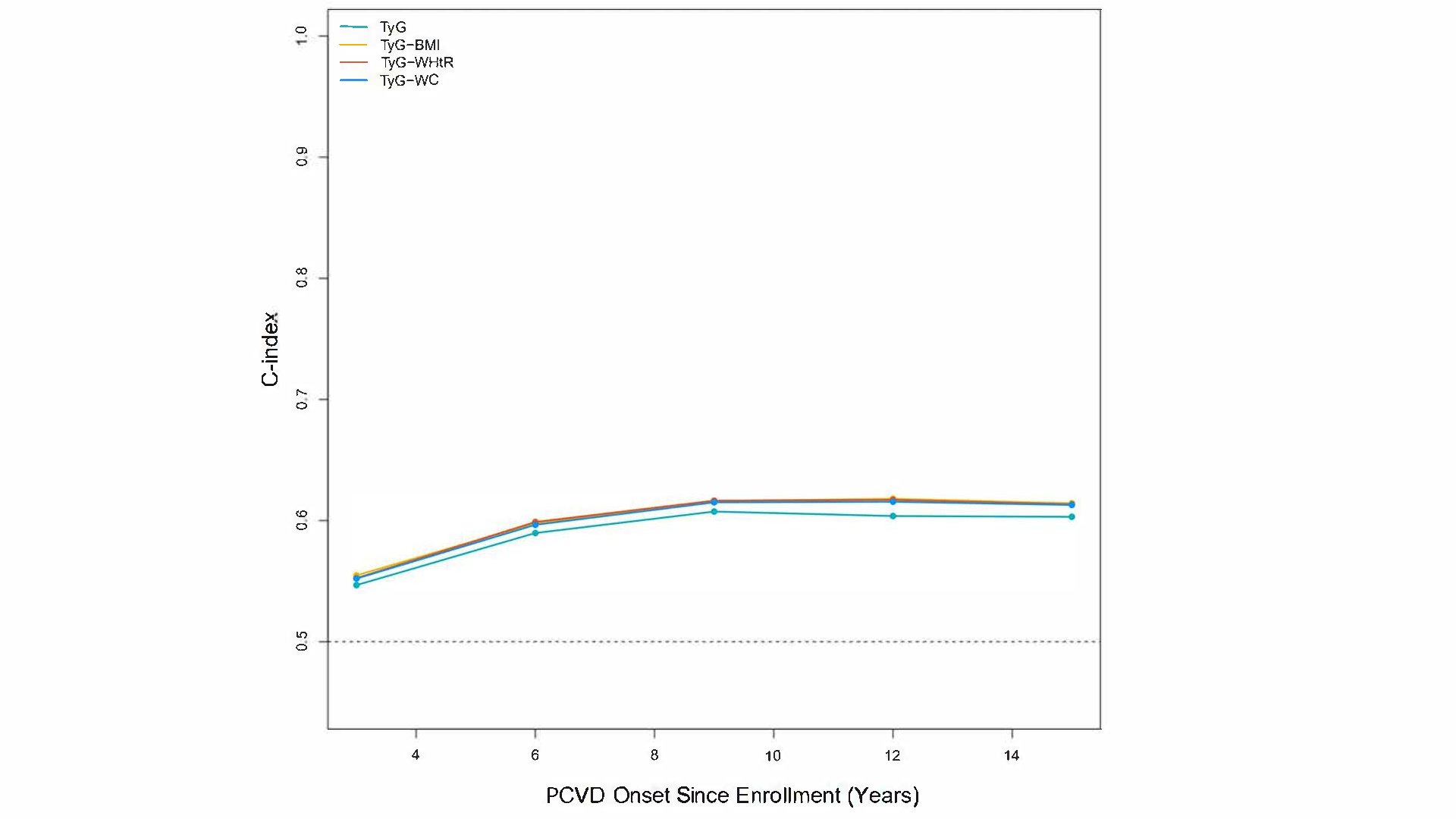

Supplement: Supplementary file 8 — Supplementary file7 Comparative predictive performance of TyG indices for postpartum cardiovascular disease. Abbreviations: BMI, body mass index; PCVD, postpartum cardiovascular disease; TyG, triglyceride-glucose; WHtR, waist-to-height ratio; WC, waist circumference. Note: Time-dependent Harrell's C-indices for cardiovascular disease prediction using TyG index and its modified indices among women with prior hypertensive disorders of pregnancy. (TIF 327 KB) [file 13167_2026_437_MOESM7_ESM.tif]

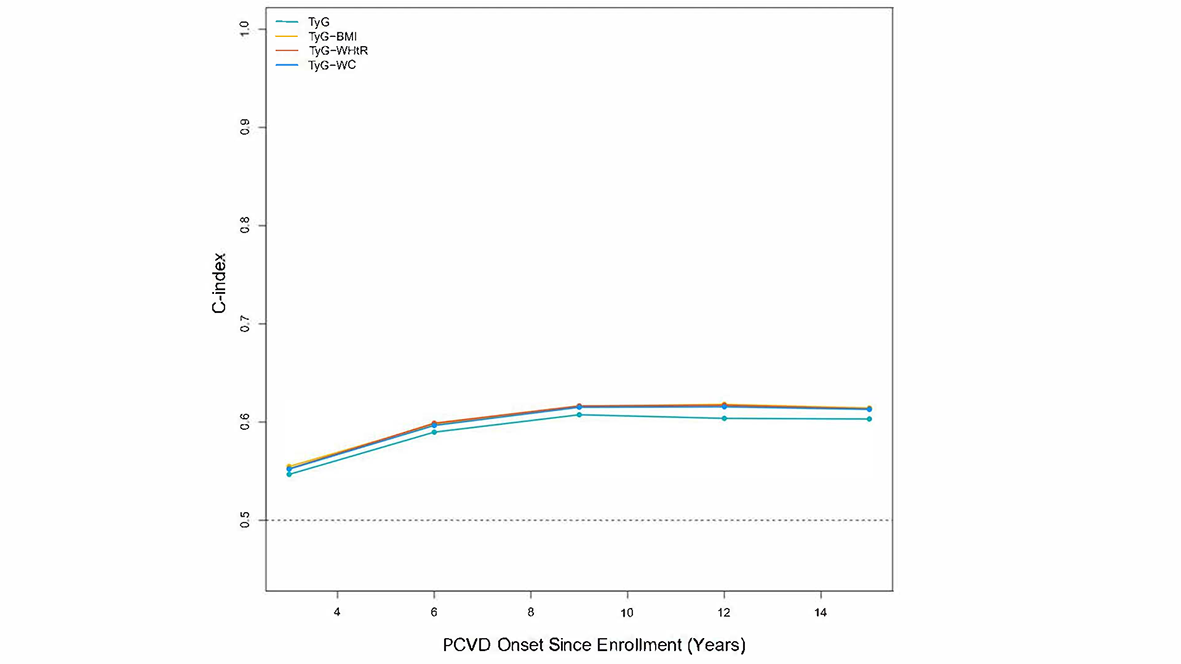

Supplement: Supplementary file 9 — (PNG 66.8 KB) [file 13167_2026_437_Fig5_ESM.png]

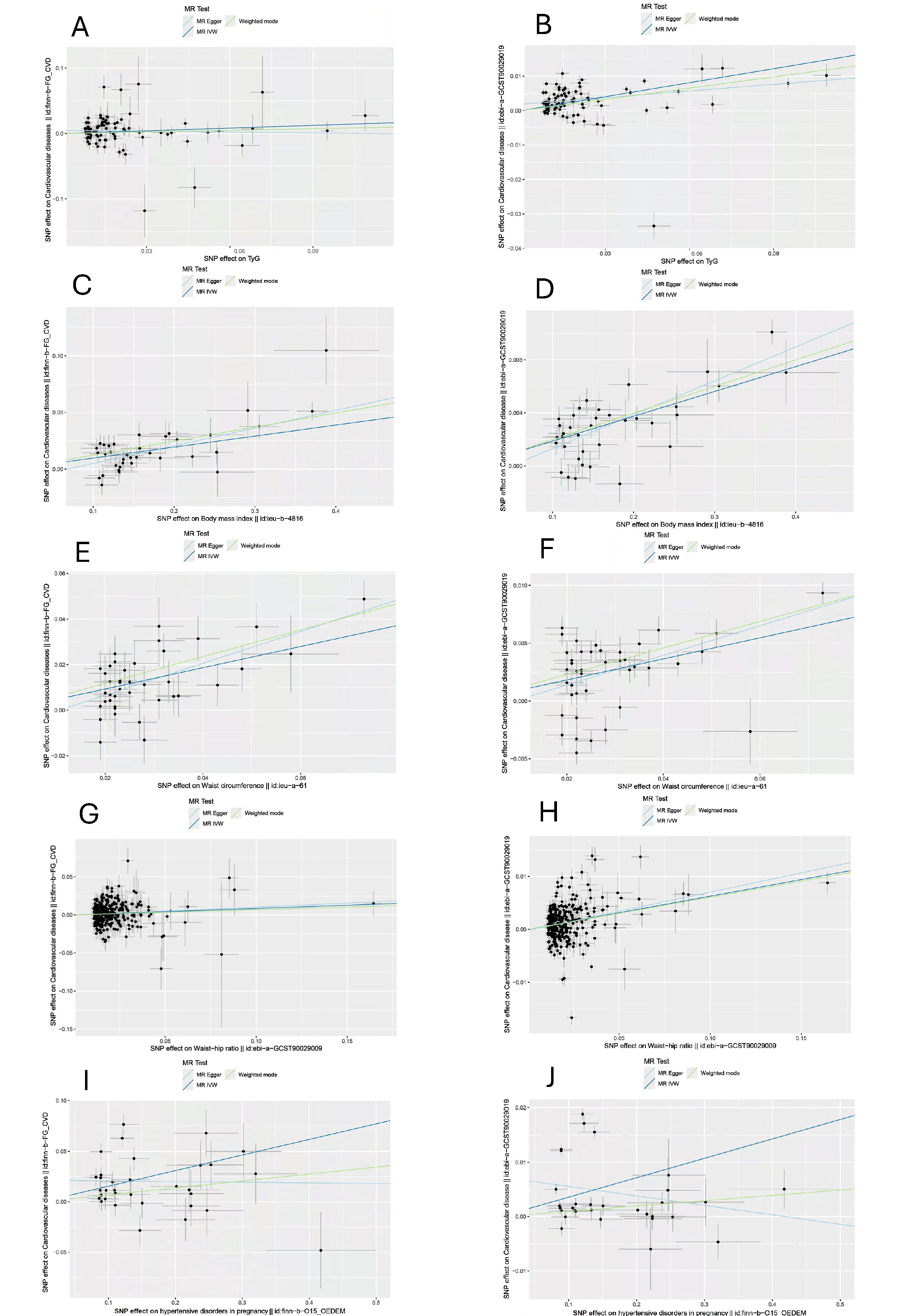

Supplement: Supplementary file 10 — Supplementary file8 The scatter plot for the Mendelian randomization analyses of causal associations. (TIFF 5876 KB) [file 13167_2026_437_MOESM8_ESM.tiff]

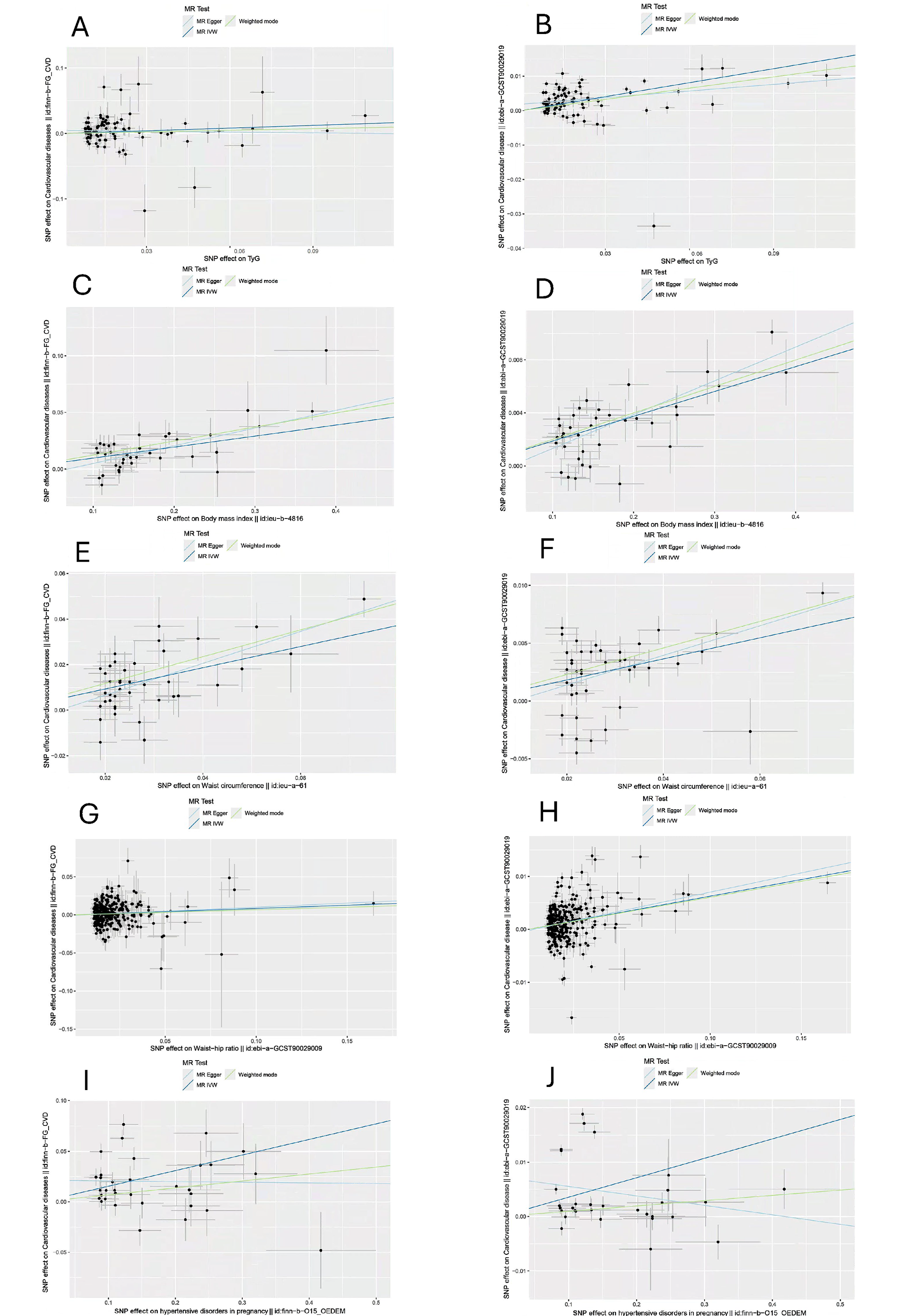

Supplement: Supplementary file 11 — (PNG 1.79 MB) [file 13167_2026_437_Fig6_ESM.png]

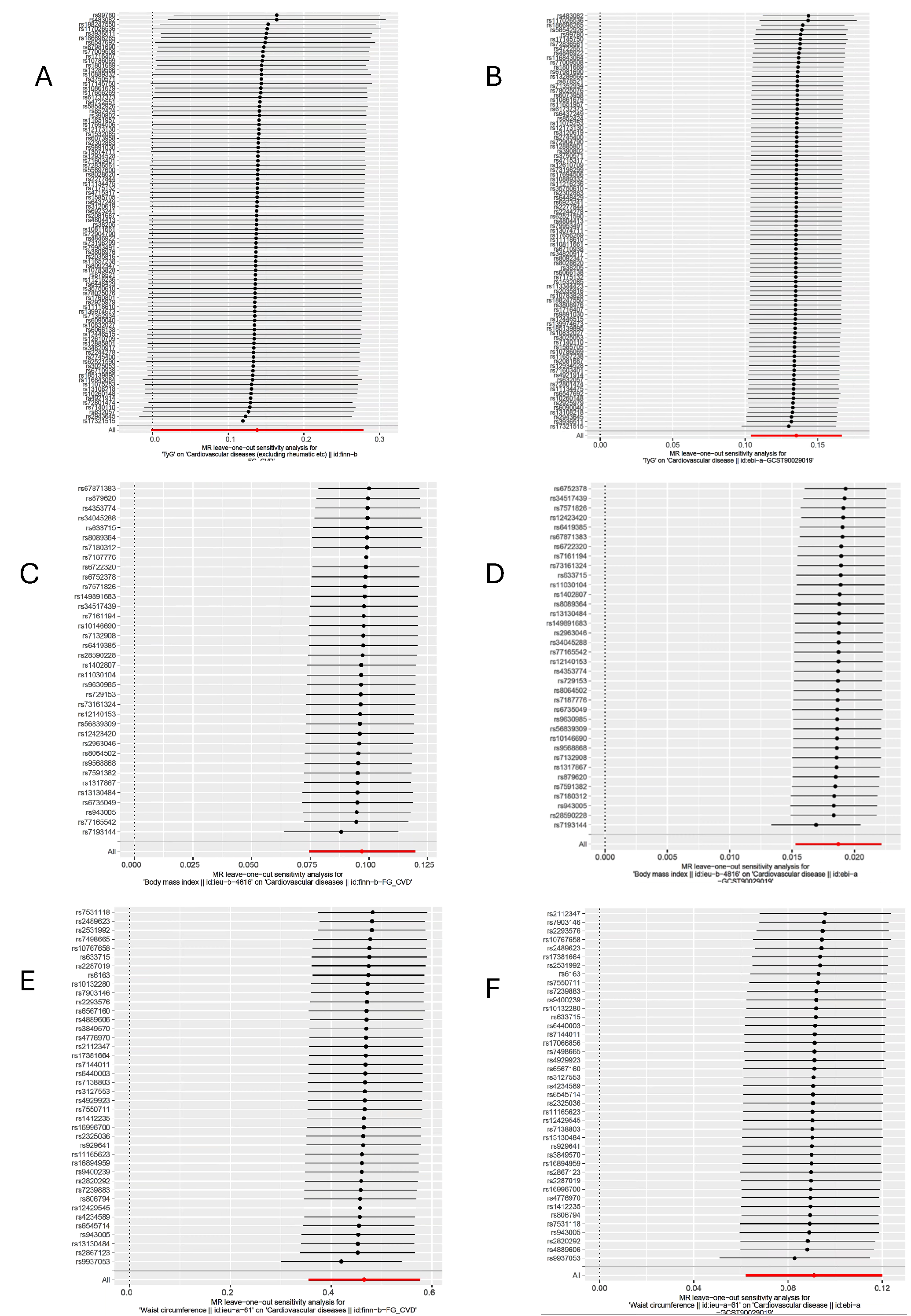

Supplement: Supplementary file 12 — Supplementary file9 Leave-one-out sensitivity analysis (TIFF 2455 KB) [file 13167_2026_437_MOESM9_ESM.tiff]

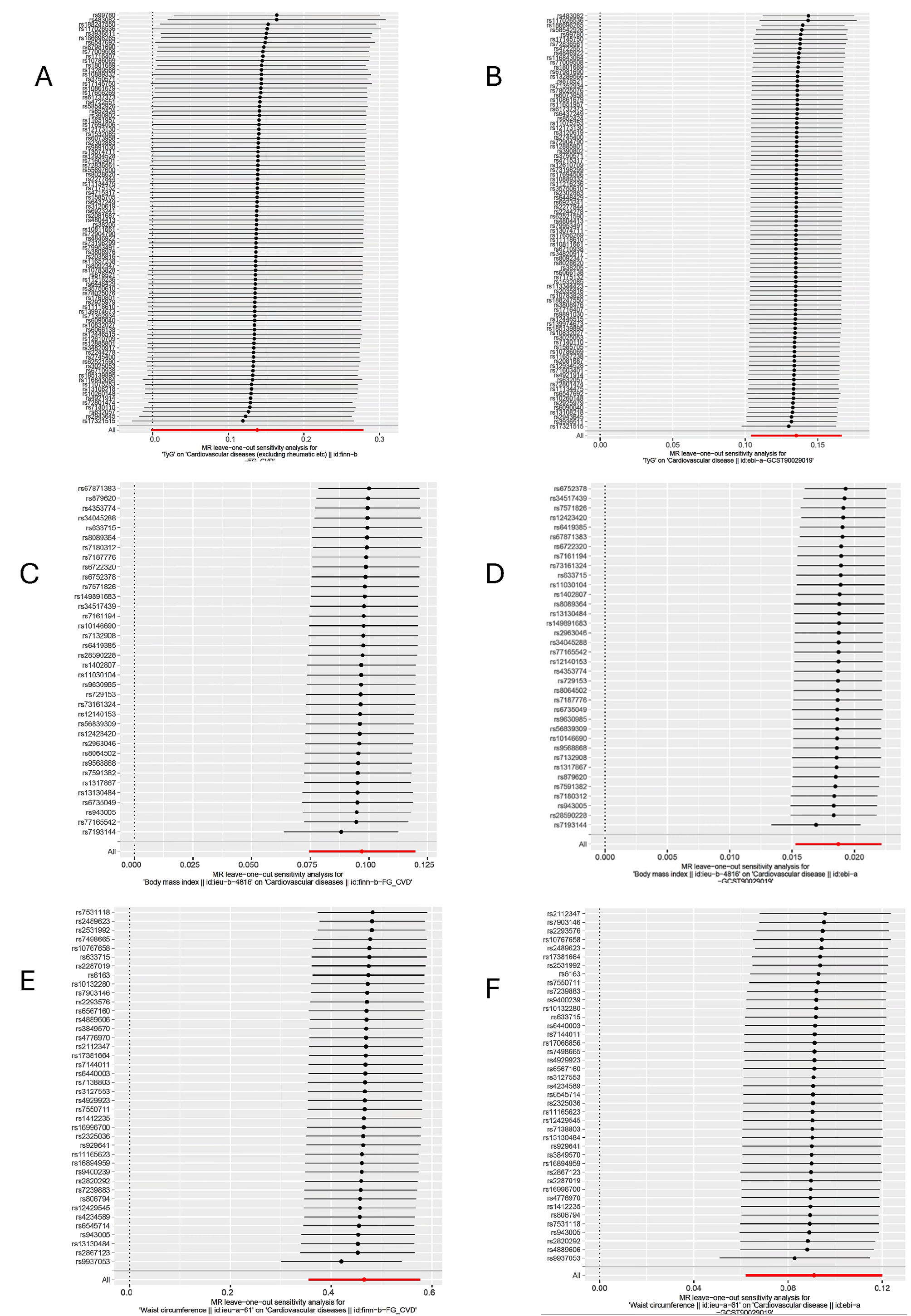

Supplement: Supplementary file 13 — (PNG 1.63 MB) [file 13167_2026_437_Fig7_ESM.png]

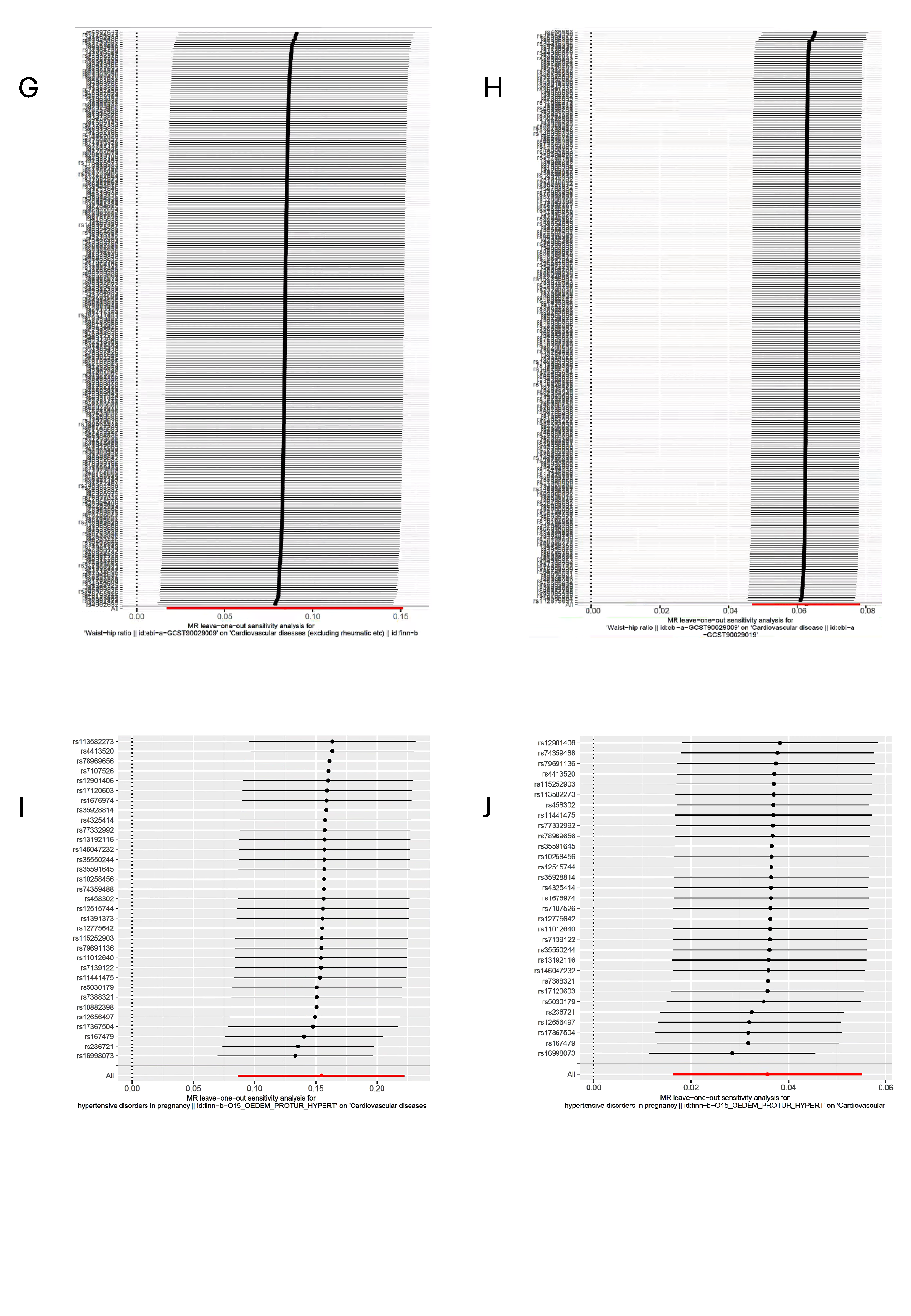

Supplement: Supplementary file 14 — Supplementary file10 (TIFF 2408 KB) [file 13167_2026_437_MOESM10_ESM.tiff]

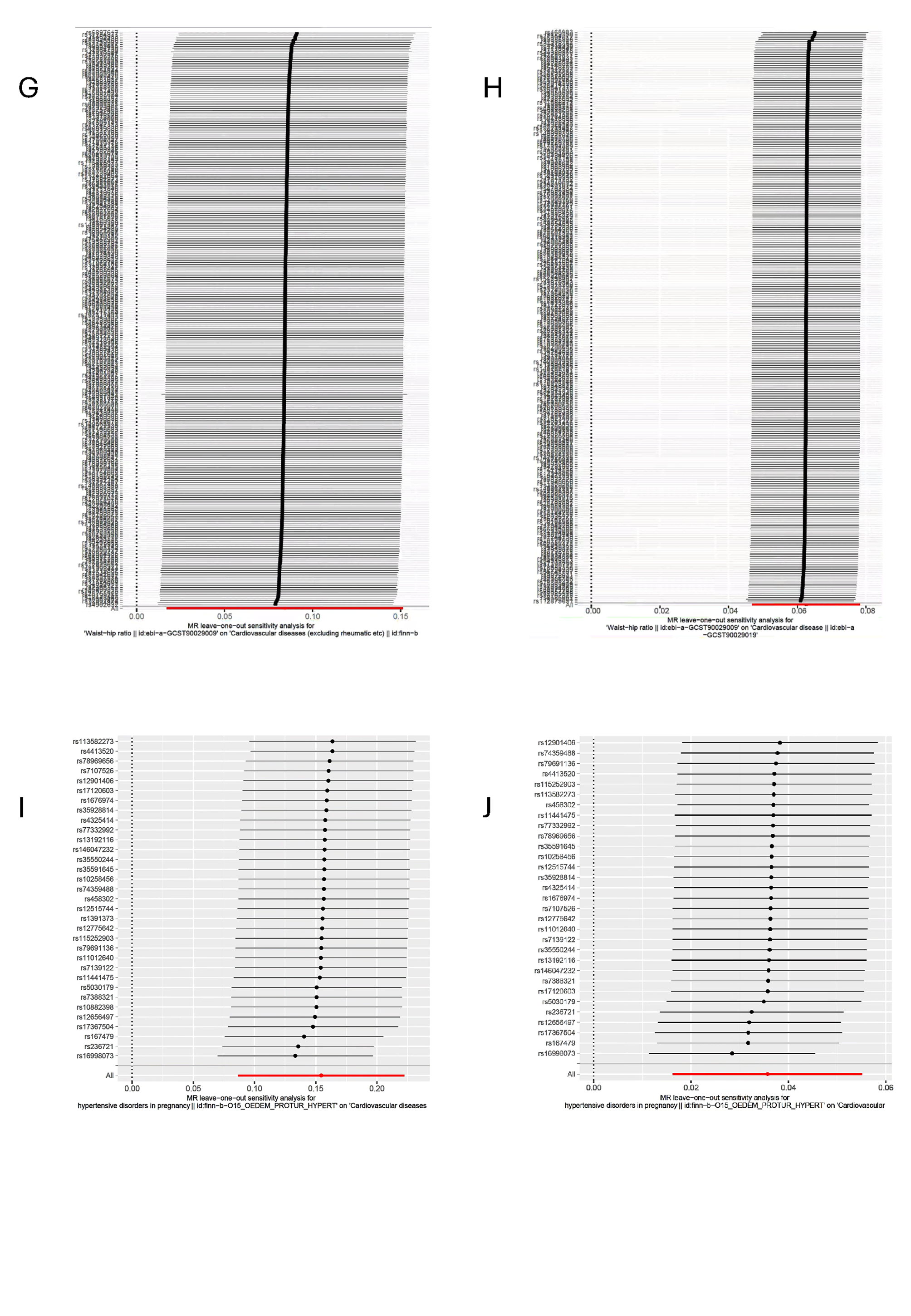

Supplement: Supplementary file 15 — (PNG 1.82 MB) [file 13167_2026_437_Fig8_ESM.png]
